# The long noncoding RNA *HORAS5* mediates castration‐resistant prostate cancer survival by activating the androgen receptor transcriptional program

**DOI:** 10.1002/1878-0261.12471

**Published:** 2019-03-05

**Authors:** Abhijit Parolia, Erik Venalainen, Hui Xue, Rebecca Mather, Dong Lin, Rebecca Wu, Perla Pucci, Jason Rogalski, Joseph R. Evans, Felix Feng, Colin C. Collins, Yuzhuo Wang, Francesco Crea

**Affiliations:** ^1^ Department of Pathology University of Michigan Ann Arbor MI USA; ^2^ British Columbia Cancer Research Centre Vancouver Canada; ^3^ Vancouver Prostate Centre Canada; ^4^ School of Life Health and Chemical Sciences The Open University Milton Keynes UK; ^5^ Proteomics Core Facility Centre for High‐Throughput Biology Michael Smith Laboratories University of British Columbia Vancouver Canada; ^6^ Department of Radiation Oncology University of Michigan Ann Arbor MI USA

**Keywords:** androgen independence, HORAS, HORAS5, lncRNA, prostate cancer

## Abstract

Prostate cancer (PCa) is driven by the androgen receptor (AR)‐signaling axis. Hormonal therapy often mitigates PCa progression, but a notable number of cases progress to castration‐resistant PCa (CRPC). CRPC retains AR activity and is incurable. Long noncoding RNA (lncRNA) represent an uncharted region of the transcriptome. Several lncRNA have been recently described to mediate oncogenic functions, suggesting that these molecules can be potential therapeutic targets. Here, we identified CRPC‐associated lncRNA by analyzing patient‐derived xenografts (PDXs) and clinical data. Subsequently, we characterized one of the CRPC‐promoting lncRNA,*HORAS5*,* in vitro* and *in vivo*. We demonstrated that *HORAS5* is a stable, cytoplasmic lncRNA that promotes CRPC proliferation and survival by maintaining AR activity under androgen‐depleted conditions. Most strikingly, knockdown of *HORAS5* causes a significant reduction in the expression of AR itself and oncogenic AR targets such as KIAA0101. Elevated expression of *HORAS5* is also associated with worse clinical outcomes in patients. Our results from *HORAS5* inhibition in *in vivo* models further confirm that *HORAS5* is a viable therapeutic target for CRPC. Thus, we posit that *HORAS5* is a novel, targetable mediator of CRPC through its essential role in the maintenance of oncogenic AR activity. Overall, this study adds to our mechanistic understanding of how lncRNA function in cancer progression.

**Abbreviations**
AR: androgen receptor
CRPC: castration‐resistant prostate cancer
DHT: dihydrotestosterone
HORAS: hormone resistance associated non‐coding sequences
HOTAIR: homeobox transcript antisense RNA
KIAA0101: PCNA clamp‐associated factor
LincRNA: long intergenic noncoding RNA
LncRNA: long noncoding RNA
LOC283070: uncharacterized long noncoding RNA
LTL: living tumor laboratory
Mir‐645: miRNA 645
NOD/SCID: nonobese diabetic/severe combined immunodeficiency
ORF: open reading frame
PCa: prostate cancer
PCAT4: prostate cancer‐associated transcript 4
PDX: patient‐derived xenograft
PSA: prostate‐specific antigen
qPCR: quantitative PCR
siRNA: small‐interference RNA
STMN1: stathmin

## 1 Introduction

Prostate cancer (PCa) proliferation is fueled by activation of the androgen receptor (AR)‐signaling pathway (Culig and Santer, 2014). Androgens directly bind to the AR and trigger this process. Thus, hormone‐deprivation therapy (aka surgical or medical ‘castration’) is an effective therapeutic strategy for localized and metastatic PCa. Unfortunately, a substantial fraction of prostatic neoplasms (~ 25%) develop resistance to castration, mainly via genetic and/or epigenetic alterations that enable aberrant ligand‐independent activation of AR‐signaling (Feldman and Feldman, 2001; Scher and Sawyers, 2005) and cell survival pathways (Gao *et al*., 2013; Karantanos *et al*., 2013). Despite the development of new therapies that delay disease progression, castration‐resistant PCa (CRPC) is still an incurable disease (Chandrasekar *et al*., 2015). Therefore, identification of alternative therapeutic targets is of paramount importance.

Long noncoding RNA (lncRNA) are non‐protein‐coding transcripts longer than 200 nucleotides. The human genome encodes for more than 50 000 unique lncRNA, most of which are uncharacterized (Iyer *et al*., 2015). In addition to their physiological roles (Li and Chang, 2014), lncRNA are involved in pathological states including cancer (Huarte, 2015; Jariwala and Sarkar, 2016). For these reasons, lncRNA have been proposed as a ‘gold mine’ for the discovery of therapeutic targets in oncology. We recently identified prostate cancer‐associated transcript 18, a lncRNA that drives androgen‐dependent PCa proliferation and metastasis (Crea *et al*., 2014) and myocardial infarction‐associated transcript, a neuroendocrine PCa‐specific lncRNA (Crea *et al*., 2016). LncRNA have been also implicated in PCa drug sensitivity (Malek *et al*., 2014), metastatic progression (Shen *et al*., 2015), and prognosis (Lee *et al*., 2014). Despite these developments, the role of lncRNA in hormonal therapy resistance has not been investigated systematically.

Patient‐derived PCa tissue xenografts (PDXs) have been very useful for translational cancer research (Hidalgo *et al*., 2014). Unlike oligoclonal cell line xenografts, PDXs more closely resemble the complex cellular heterogeneity of human PCa (Siolas and Hannon, 2013). We have developed a unique procedure for grafting and serially transplanting primary human cancer tissues in nonobese diabetic/severe combined immunodeficiency (NOD/SCID) mice, using the subrenal capsule graft site (Lin *et al*., 2014b). These transplantable PDXs accurately recapitulate donor patient's tissue histology, genetic/epigenetic features, and drug sensitivity (Lin *et al*., 2014a). We have successfully applied PDXs for drug efficacy studies, discovery and validation of therapeutic targets, and personalized cancer therapy (Lin *et al*., 2014a).

Using this technique, we have recently developed pairs of PDXs with opposite sensitivity to castration (Lin *et al*., 2014a). Here, we profiled three of these pairs to identify lncRNA specifically up‐regulated in metastatic CRPC. Our results indicate that at least one of these previously uncharacterized transcripts activates pro‐survival pathways and is functionally relevant in the progression of PCa to a castration‐resistant state.

## 2 Materials and methods

### 2.1 Cell culture

Human PCa cell lines were purchased from the American Type Culture Collection (ATCC, Burlington, ON, Canada). Cells were cultured in RPMI‐1640 media (Gibco, Burlington, ON, Canada, Cat# 11875‐093) supplemented with 10% FBS (Gibco, Cat# 10099‐141) and following ATCC protocols for culture passage and storage of cells (ATCC, cryogenic storage of animal cells protocol). A humidified 37 °C 5% CO_2_ incubator was employed for all culturing. Genetic fingerprinting and monthly Mycoplasma tests were conducted at the Vancouver Prostate Centre. Unless otherwise stated, all cells were counted for experiments using a TC20 automated cell counter (Bio‐Rad, Mississauga, ON, Canada, Cat# 1450102) following manufacturer's protocol (TC20 automated cell counter quick guide) and were maintained until a passage of 20 or lower.

### 2.2 Patient‐derived PCa xenograft

For generation of PDX models, primary tumor biopsies were collected at the BC Cancer Agency or the Vancouver Prostate Centre with patients’ written consent. All surgical procedures and protocols for the acquisition and research‐wise handling of tumor biopsies were approved by The University of British Columbia (UBC)—Research Ethics Board (protocol#: H04‐60131). PDX was maintained by transplantation into subrenal capsules of male NOD‐SCID mice obtained from the British Columbia Cancer Research Centre—Animal Resource Centre (Vancouver, Canada), as previously described (Lin *et al*., 2014a). All animal experiments were performed following the ethical guidelines and standards set by the Declaration of Helsinki and in accordance with the established animal care and use protocols approved by the UBC Animal Care Committee (protocol #: A10‐0100).

### 2.3 RNA extraction, reverse‐transcription, and quantitative PCR (qPCR)

Total RNA was extracted using the RNeasy Mini Kit (Qiagen, Toronto, ON, Canada) from cultured cells or tissues following manufacturer's protocol. Upon extraction, 1000 ng of total RNA was reverse‐transcribed using the QuantiTect kit (Qiagen) following manufacturer's protocol. Reverse‐transcription, genomic DNA digestion was performed prior to cDNA synthesis following manufacturer's instructions. Predesigned or custom TaqMan primers (Life Technologies, Burlington, ON, Canada, Assay IDs for all probes listed in Table S6) were used for quantitative PCR (qPCR) to assess gene expression as per manufacturer's protocol. Subcellular RNA fractionation was performed using the Paris kit (Ambion, Life Technologies) following the manufacturer's protocol and performing the optional nuclear pellet washing step to obtain purer fractions.

### 2.4 siRNA‐mediated gene knockdown

Gene knockdown was performed using the reverse transfection method (Hattori *et al*., 2017). Cells were seeded in a six‐well or 96‐well plate along with the lipid:small‐interference RNA (siRNA) mixtures prepared using the RNAiMAX (Invitrogen, Burlington, ON, Canada) reagent as per the manufacturer's protocol. Final siRNA treatment dosages were 2 nm, and all duplexes were purchased from IDT: *HORAS5* (aka *Linc00161*, anti‐*HORAS5* DsiRNA 1: 5′‐GUGAUAAUAAUAUAAACUACAGUCA‐3′, anti‐*HORAS5* DsiRNA 2: 5′‐CUAUGACUGUGGUAAACAUUUCCAA‐3′), PCNA clamp‐ associated factor (*KIAA0101*; anti‐*KIAA0101* DsiRNA 1: 5′‐GUUUACCCUGGUAUUCUAGAAUGTA‐3′, anti‐*KIAA0101* DsiRNA 2: 5′‐AGUGUCUAGUUCUUGCUAAAAUCAA‐3′), and nontargeting negative control, Cat#: 123762010. After 48 or 72 h post‐transfection, treated cells were harvested for extracting total RNA and/or total protein.

### 2.5 Cell proliferation and caspase activity assays

For both LNCaP and C4‐2, 2000 viable cells were seeded in a 96‐well plate and reverse transfected with either 2 nm of control siRNA or siRNA targeting the *HORAS5* transcript with six replicates for each treatment. At days 1, 3, 5, and 7 post‐transfection, cell viability was assessed using the colorimetric CellTiter 96 Aqueous One Solution Cell Proliferation Assay (Promega, Madison, WI, USA, Cat# G3582) according to the manufacturer's protocol. Wells were incubated at 37 °C in a 5% CO_2_ incubator for 1.5 h prior to absorbance measurements using a spectrophotometer set to 490 nm. Values were plotted for each treatment after normalizing to the NC Day 1 average reading.

#### 2.5.1 DHT‐rescue experiment

A total of 2000 viable LNCaP cells per well were plated in a 96‐well plate and reverse transfected with *HORAS5*‐targeted or control siRNA (2 nm dosage) as described above. Cells were then daily supplemented with either 10 nm dihydrotestosterone (DHT) or ethanol (vehicle control), and viability was calculated using the MTS assay and described above.

#### 2.5.2 Caspase activity assays

Cells were plated in a white, flat‐bottom 96‐well plate and treated with siRNA as described with 6 replicates for each treatment. At day 3 post‐transfection, Caspase‐Glo reagent (Promega) was added to cells and total luminescence was quantified following manufacturer's protocol. These luminescence values were normalized to time‐match and treatment‐matched cell viabilities for each group and were plotted relative to the control siRNA‐treated samples.

### 2.6 RNA half‐life measurement

A total of 100 000 cells were plated in a six‐well and treated with 50 μg·mL^−1^ of alpha‐amanitin (Sigma, Oakville, ON, Canada, A2263), as described (Khalil *et al*., 2008). Total RNA was extracted at time‐points 0, 2, 5, 6, and 10 h postincubation, and gene expression was analyzed by qPCR as previously described.

### 2.7 Cell cycle analysis

Cell cycle phase estimates were obtained using fluorescence‐activated cell sorting flow cytometry. First, LNCaP and C4‐2 cells were treated with a scramble control duplex or targeted anti‐*HORAS5* DsiRNA for 72 h. A total of 4.0 × 10^5^ cells were then harvested post‐treatment, including untreated and lipofectamine RNAiMAX controls, and resuspended in 100 μL of ice‐cold DPBS (Gibco, Cat# 14190144). Next, 900 μL of ice‐cold 70% ethanol was added dropwise while vortexing cells to prevent aggregates from forming. Cells were left in 15‐mL falcon tubes at 4 °C for 1 week for fixation. The following week, the ethanol was removed by centrifugation and cells were washed with DPBS once. Cell cycle phase was determined using propidium iodide (PI) staining buffer of composition 0.1% Triton X‐100, 100 μg·mL^−1^ RNase (Invitrogen, Cat# 12091039), and 10 μg·mL^−1^ of PI (Sigma, Cat# P4170). After 30 min in the dark with the PI stain, cells were analyzed by FACScalibur (Becton Dickenson, Mississauga, ON, Canada) and were gated first by side scatter vs forward scatter, and then by FL2‐Area vs FL2‐Width. Counts were then plotted against FL2‐Area for selection of G1, S, and G2/M phases. Phasing estimation was obtained using flowjo v10 software (FlowJo, Ashland, OR, USA). A total of 20 000 cells were analyzed to get representative counts per phase. Significance was determined by comparing the NC to treated cells in each phase (Student's *t*‐test). ‘Cell cycle phase’ represents either a single (G1) or double (S/G2‐M) complement of DNA.

### 2.8 Western blotting analysis

Cell lysates were created using 100 μL of RIPA buffer unless otherwise stated. Proteins (50 μg) were resolved via gel electrophoresis on reducing SDS/PAGE run at 100V for 1 h. Transfer to nitrocellulose membranes was done at 100V for 1 h as well. The membranes were blocked in 5% skim milk dissolved in TBS‐T wash buffer (Tris‐buffered saline containing 0.1% Tween) at room temperature for 1 h. Following this, blots were incubated overnight at 4 °C with protein‐specific primary antibodies containing 5% BSA to β‐actin at 1 : 5000 (Sigma, Cat# A5441), KIAA0101 at 1 : 1000 (Abnova, Burlington, ON, Canada, Cat# H00009768‐M01), and AR Primary Ab (Santa Cruz, Mississauga, ON, Canada, Cat# sc‐7305) 1 : 500 in TBS‐T + 5% BSA. After incubation, blots were washed four times in TBS‐T for 5 min each. Lastly, blots were incubated with HRP‐conjugated anti‐mouse secondary antibody diluted in 10 mL of TBS‐T at room temperature for 1 h (β‐actin 1 : 5000, KIAA0101 1 : 1000, and AR 1 : 2000). After washing as above, chemiluminescence was determined in a GelDoc system using an ECL western blotting substrate kit (ThermoFisher, Burlington, ON, Canada Cat# 32016).

### 2.9 *In vivo* gene knockdown

Gene knockdown *in vivo* was achieved using the AteloGene *in vivo* siRNA/miRNA Transfection kit (REPROCELL, Beltsville, MD, USA) following manufacturer's protocol. Castrate‐resistant LNCaP cells were xenografted in both dorsal flanks of intact male, immunocompromised NOD/SCID mice as previously described (Kuruma *et al*., 2013) and were randomly assigned to each treatment group (*n* = 3, i.e., six tumors/treatment). Two nanomolar total dosage of *HORAS5* siRNA was locally injected to surround the tumor as per manufacturer's protocol for two consecutive days. Tumors were harvested 3 days after the first injection to assess tumor volume and target knockdown. All animal experiments were performed following the ethical guidelines and standards set by the Declaration of Helsinki and in accordance with the established animal care and use protocols approved by the UBC Animal Care Committee (protocol #: A10‐0100). Total RNA was extracted as described above, and genes were quantified using RT‐qPCR to assess knockdown efficiency.

### 2.10 Wound‐healing (migration) assays

For LNCaP and C4‐2 cell lines, 18 h post‐transfection with either NC duplex or anti‐*HORAS5* siRNA. 2.0 × 10^6^ cells per six well for LNCaP cells, and 3.5 × 10^6^ cells per 60‐mm dish for C4‐2 cells were seeded. Only six wells were precoated with poly‐l‐lysine for 3 h (Sigma, Cat# P4707) and rinsed with dH_2_O. Both LNCaP and C4‐2 cells were left to adhere for 4–5 h before scratching the wells using a sterile P10 micropipette tip. Assays used serum‐free media to prevent cell growth. Twenty‐four hours postscratch, cells were visualized and captured using an AxioCam MRc real‐time camera (Zeiss, Toronto, ON, Canada). Wound closure was quantified using Adobe Photoshop as described (Chiang *et al*., 2014).

### 2.11 Boyden chamber (invasion) assays

LNCaP and C4‐2 cells were first either left untreated, or were transfected with NC duplex or anti‐*HORAS5* siRNA for 18 h in 60‐mm dishes. Next, cell invasive potential was examined using polyethylene terephthalate‐coated 24‐well transwell chambers with a pore size of 8 μm (Corning, Corning, NY, USA, Cat# 354480). Prior to the assay, top and bottom chambers were re‐hydrated using 500 μL of prewarmed serum‐free media for 2 h at 37 °C. Following this, 1.0 × 10^5^ cells were seeded per well in serum‐free media, and the assay was carried out according to manufacturer's protocol. Media supplemented with 10% FBS was used as a chemoattractant in the bottom of each well. Cells were incubated for 48 h prior to quantification using cell dissociation solution (Trevigen, Gaithersburg, MD, USA, Cat# 3455‐096‐05) diluted in MQH_2_O and Calcein AM (Trevigen, Cat# 4892‐010‐01). Once mixed, 300 μL of complete dissociation solution was added to the bottom chambers for 1 h. One hundred microliter of dissociated cells was used for 96‐well plate quantification using an excitation and emission of 485 and 520 nm, respectively. All readings were done in duplicate.

### 2.12 Immunohistochemical staining

*In vivo* treated tumors from all groups were used to generate tissue microarray and stained for cleaved Caspase 3 using a rabbit monoclonal antibody at 1 : 50 dilution (Cell Signaling, Whitby, ON, Canada, Cat# 9664). Tissue sections were divided into nine quadrants prior to counting. Five of the total nine quadrants were used to take positively stained cell counts, which were then compared to total cells in the visual field. All five field counts were averaged and converted to a percentage value for control and treatment.

### 2.13 RNA sequencing and differential expression analysis

Total RNA was used to generate the sequencing library as previously described (Lin *et al*., 2014a). Briefly, RNA sequencing fastq files were processed to read counts using the STAR short read aligner (https://github.com/alexdobin/STAR) with an index built with the hg19 genome GENCODE v19 transcriptome reference, and the ‐quantMode option. Genes with 0 counts in both libraries were eliminated. Twenty‐five (25) was added to the read count for each gene to handle genes with 0 reads in one library and normalized per million mapped reads (RPKM) in each library. Genes were then filtered for ‘lincRNA’ type and sorted in descending order of up‐regulation in the CRPC [living tumor laboratory (LTL)‐313BR] sample. To identify the up‐ and down‐regulated lincRNA, fold‐change (FC) cutoff of three was used and lincRNA were ranked in order of FC for hormone resistance associated non‐coding sequences (*HORAS*) annotation.

### 2.14 Proteomics analysis

A total of 10 million viable LNCaP cells were plated in a T150 flask and treated with either control or anti‐*HORAS5* as described earlier. At 3 days after treatment, cells were washed once with ice‐cold 1× PBS and snap‐frozen to perform the proteomics analysis at the UBC Proteomics Core Facility at the Centre for High‐Throughput Biology (Vancouver, Canada). Briefly, protein extracts from treated cells were suspended in SDS sample buffer and run on a short 10% SDS/PAGE gel. Proteins were visualized by colloidal coomassie (Candiano *et al*., 2004) and digested out of the gel as described (Chan *et al*., 2006). Peptide samples were purified by solid phase extraction on C‐18 stop and go extraction (STAGE) Tips (Ishihama *et al*., 2002), and each treatment was labeled by reductive dimethylation using formaldehyde isotopologues (Parker *et al*., 2012). The final product was purified again by C18 STAGE tips as previously done and analyzed by LC‐MSMS (Kang *et al*., 2014). Protein identification and quantification were performed with maxquant v1.5.1.0 as described (Cox *et al*., 2009).

### 2.15 *In silico* miRNA binding prediction analyses

In order to predict miRNA that could be interacting with *HORAS5*, we used an online transcriptome‐wide miRNA binding prediction tool (http://www.mircode.org/index.php) from the Larsson Lekholm Lab from the Institute of Biomedicine, University of Gothenburg (http://larssonlab.org/). The tool was run using default parameters, and the entire output data have been summarized in a table in Fig. S12. Additional information about this prediction software is available on the lab's website.

### 2.16 Availability of data and materials

All data generated or analyzed during this study are included in this published article (and its supplementary information files).

## 3 Results

### 3.1 Identification of hormone therapy resistance‐associated lincRNA

**Figure 1:**
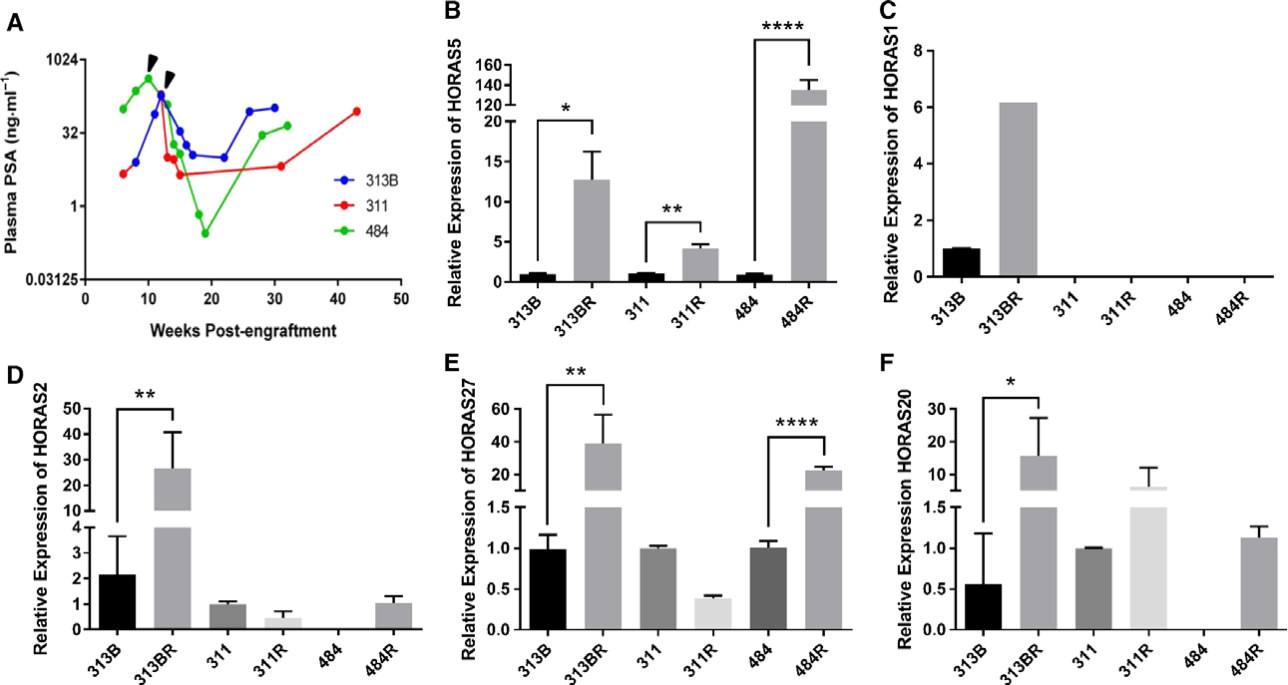
*HORAS5* is up‐regulated in CRPC. (A) PSA levels in three independent hormone‐sensitive patient‐derived xenografts in NOD‐SCID mice. Arrows represent androgen deprivation (via surgical castration) followed by relapse around 20–30 weeks. (B–F) qPCR expression of *HORAS* *5* (B, aka *linc00161*), *HORAS1* (C, aka *RP11‐945A11.1*), *HORAS2* (D, aka *AF131217.1*), *HORAS27* (E, aka *PCAT4*), *HORAS20* (F, aka *AP001604.3*) identified from differential expression analyses between hormone‐sensitive (LTL313B) and relapsed (LTL313BR) PCa PDXs. Results expressed as means ± SD from two independent replicates. Student's *t*‐test was performed for statistical comparisons. *P < 0.05, **P < 0.01, and ****P <  0.0001. *HORAS1* was undetectable in the remaining samples.

To identify lncRNA that are up‐regulated upon progression of primary PCa to CRPC, we analyzed our unique collection of PCa PDX models (Lin *et al*., 2014a). These PDXs were grown in the subrenal capsule of immunocompromised male mice. PDX‐bearing mice were surgically castrated at about 10–12 weeks postengraftment (black arrowheads, Fig. 1A). As expected, castration resulted in a dramatic reduction of plasma prostate‐specific antigen (PSA) levels, which is a canonical AR target gene and strongly correlated with the total tumor burden (Figs 1A and S1A). Analogous to the clinical progression of some PCas, in three PDX models the tumors eventually relapsed after about 20 weeks to produce a castration‐resistant subline. The relapsed, castration‐resistant tumors displayed signs of AR‐signaling re‐activation, as evidenced by the parallel increase in plasma PSA levels (Figs 1A and S1A). Of these PDX models, we chose the most well‐established and previously described LTL‐313B/BR pair for a comprehensive RNA sequencing analysis (Luk *et al*., 2017). Notably, the relapsed castration‐resistant LTL‐313BR subline retains AR expression and is markedly resistant to clinical AR‐antagonistic drugs bicalutamide and enzalutamide relative to the parental subline (Luk *et al*., 2017). This implies that a broad spectrum of molecular mechanisms that regulate CRPC growth underlies the survival and proliferation of LTL‐313BR under castrate conditions. To investigate this further, we decided to focus on the intergenic subclass of lncRNA (hereinafter referred to as lincRNA) that are coded on distinct chromosomal loci and thus are easier to functionally characterize. Differential gene expression analysis between LTL‐313BR vs 313B revealed 57 lincRNA that were up‐regulated (FC ≥ 3), and 329 lincRNA that were down‐regulated (FC ≤ −3) in the hormone therapy‐resistant subline (Tables S1 and S2, respectively). Consistent with previous studies, we found PCa progression‐associated *PCAT4* (Prensner *et al*., 2011) among the up‐regulated transcripts, and *in vivo* androgen‐regulated prostate cancer‐associated transcript 9 (Parolia *et al*., 2015) among the down‐regulated lincRNA. Since a relevant portion of these lincRNA were previously unannotated, we termed them as *HORAS*. To confirm the RNA sequencing results and to profile the expression of other differentially expressed lincRNA in other PDX pairs, we performed qPCR gene expression analyses. Our results confirmed up‐regulation of the selected *HORAS* transcripts in LTL313BR vs LTL‐313B, and in two other PDX pairs (Fig. 1B–F). *HORAS5* (*aka linc00161, NCRNA00161, C21orf100, linc‐USP16*) was significantly up‐regulated in all the three castration‐resistant PDXs (4–130‐fold; Fig. 1B). Therefore, we pursued *HORAS5* for further functional characterization in CRPC‐derived PCa cells.

The Ensembl database categorizes *HORAS5* as a lincRNA gene and annotates it as *linc00161* (aka *C21orf100, NCRNA00161*) with two splice variants. The open reading frame (ORF) finder database (http://www.ncbi.nlm.nih.gov/gorf/gorf.html) confirmed that at least 88% of this transcript does not contain an ORF. Test code software (Fickett, 1982) confirmed that the transcript is noncoding (*P* < 0.01). Interestingly, *HORAS5* was first described in a study published over a decade ago which attempted to profile novel genes encoded on chromosome 21 and could be implicated in Down syndrome (Reymond *et al*., 2002). At the time, the authors confirmed *HORAS5* transcript structure via 5′‐ and 3′‐end rapid amplification of cDNA ends. Additionally, the same study showed via qPCR that *HORAS5* was robustly expressed only in normal prostate tissue compared to 20 other normal tissue types (Reymond *et al*., 2002). This prostate‐specific expression was corroborated by an independent study using an array‐based transcriptomic technique in a panel of 12 normal tissues (Fig. S2A). Concordantly, we found *HORAS5* to show the highest expression in PCa specimens across all TCGA‐sequenced cancers (Fig. S2B). Of note, a recent study reported that *HORAS5* was induced by chemotherapy in osteosarcoma cells (Wang *et al*., 2016b). However, no functional knowledge exists on the biological role of *HORAS5* in PCa pathogenesis.

**Figure 2:**
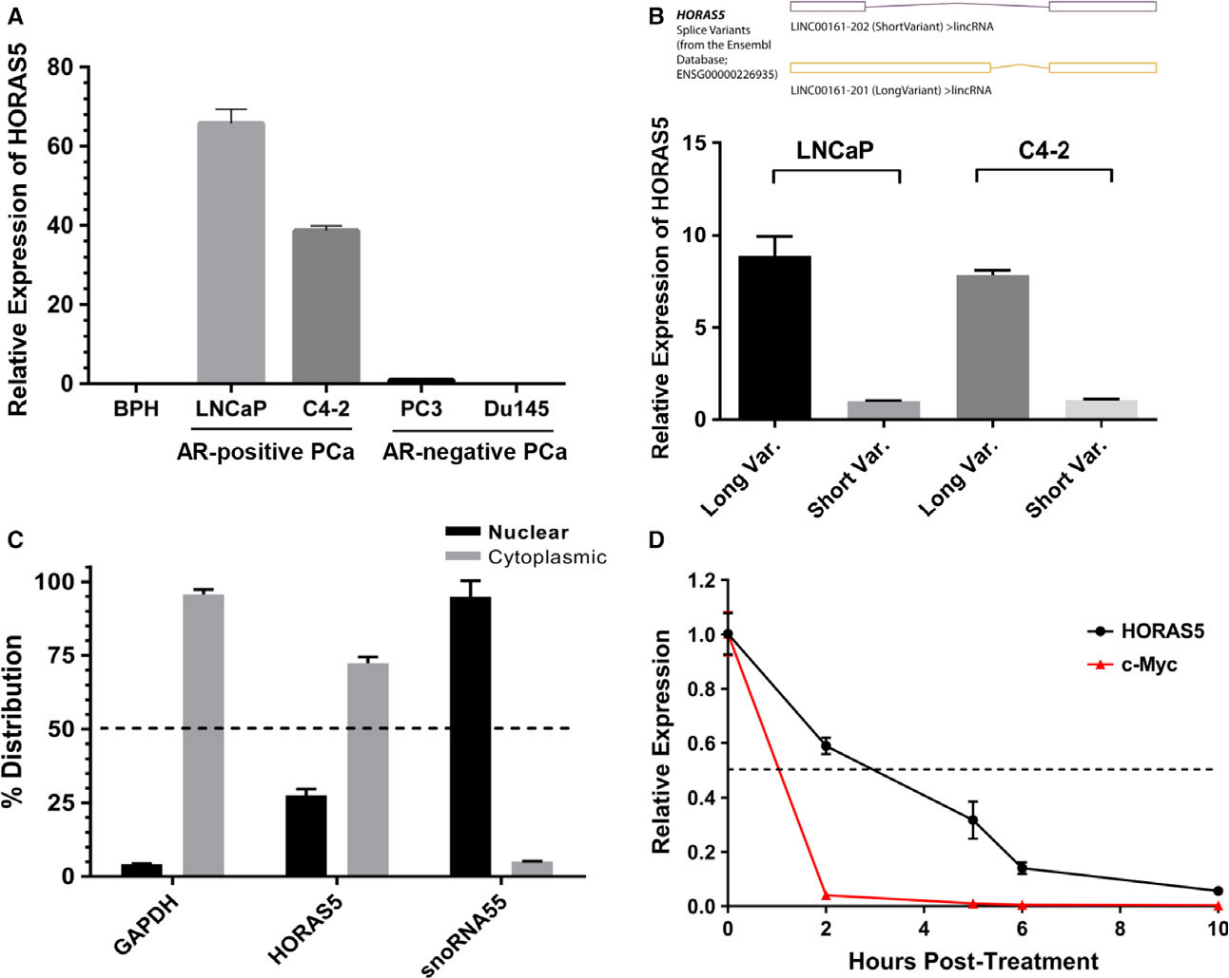
*HORAS5* is a stable cytoplasmic lncRNA in AR‐positive PCa cells. (A) Basal expression (qPCR) of *HORAS5* in a panel of PCa cell lines. Gene expression was normalized to average of *GAPDH* and *HPRT1* and shown relative to BPH1. (B) Taqman qPCR expression of *HORAS5* long and short variants (as identified in the Ensembl database) in two CRPC‐derived cell lines, namely LNCaP and C4‐2. (C) qPCR expression of *HORAS5* in the nuclear and cytoplasmic subcellular fractions of AR‐positive LNCaP cells. *GAPDH* and *snoRNA55* are used as controls for cytoplasmic and nuclear fractions, respectively. Results from two independent experiments are plotted. (D) qPCR‐based transcript expression of *HORAS5* and c‐Myc in LNCaP cells treated with alpha‐amanitin for various time‐points (0, 2, 5, 6, 10 h post‐treatment). Relatively stable GAPDH expression was used for normalization, and data are plotted relative expression at 0 h. Results from two independent experiments are plotted as means ± SD.

As a first step, we profiled the expression of *HORAS5* in a panel of PCa cell lines. We found that *HORAS5* was considerably expressed only in AR‐positive CRPC‐derived cell lines (Sedelaar and Isaacs, 2009; Thalmann *et al*., 1994), and was undetectable in non‐neoplastic BPH cells (Fig. 2A). Our data also indicated that the longer *HORAS5* transcript variant was much more abundant than the shorter one in most PCa cells and PDX models (Figs 2B and S3A,B), prompting us to functionally pursue the longer variant (hereafter referred to as *HORAS5* alone). This restricted *in vitro* expression of *HORAS5* in AR‐positive CRPC cell lines is consistent with the *in vivo* expression profile observed in our AR‐positive CRPC‐derived PDX models.

Next, subcellular RNA fractionation experiments revealed that *HORAS5* was predominantly localized in the cytoplasm of the cell (Figs 2C and S3C). Since lincRNA, unlike a protein‐coding mRNA, are end functional entities, we investigated the stability of the *HORAS5* transcript itself. To this end, we treated LNCaP cells with α‐amanitin, a potent inhibitor of RNA polymerase II, and thereafter measured the relative RNA abundance at various time‐points (qPCR). Compared to the mRNA of a known oncogenic factor, *c‐Myc*,* HORAS5* possessed a longer half‐life of about 3 h (Fig. 2D). This implies that *HORAS5* is relatively stable inside cells for several hours post‐transcription, thereby allowing it to mediate important cellular functions.

### 3.2 Functional characterization of *HORAS5*

**Figure 3:**
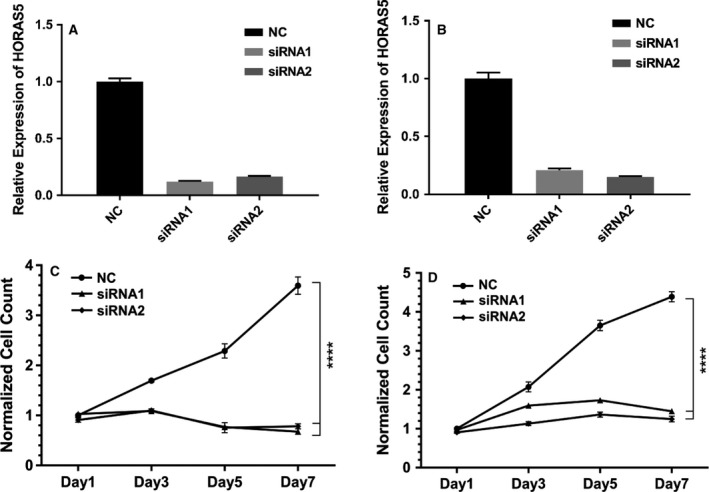
*HORAS5* knockdown attenuates cell proliferation *in vitro*. (A, B) qPCR expression levels of *HORAS5* in LNCaP (A) and C4‐2 (B) cells upon treatment with nontargeting control siRNA (NC) or two distinct anti‐*HORAS5* siRNA (2 nm dosage). Data shown are a single representative trial. (C, D) MTS cell proliferation assays for LNCaP (C) and C4‐2 (D) upon treatment with anti‐*HORAS5* siRNA or controls siRNA. Two‐way ANOVA with multiple comparisons (Tukey's post *t*‐test) performed to measure significance, ****P < 0.0001. Results are from three independent experiments.

**Figure 4:**
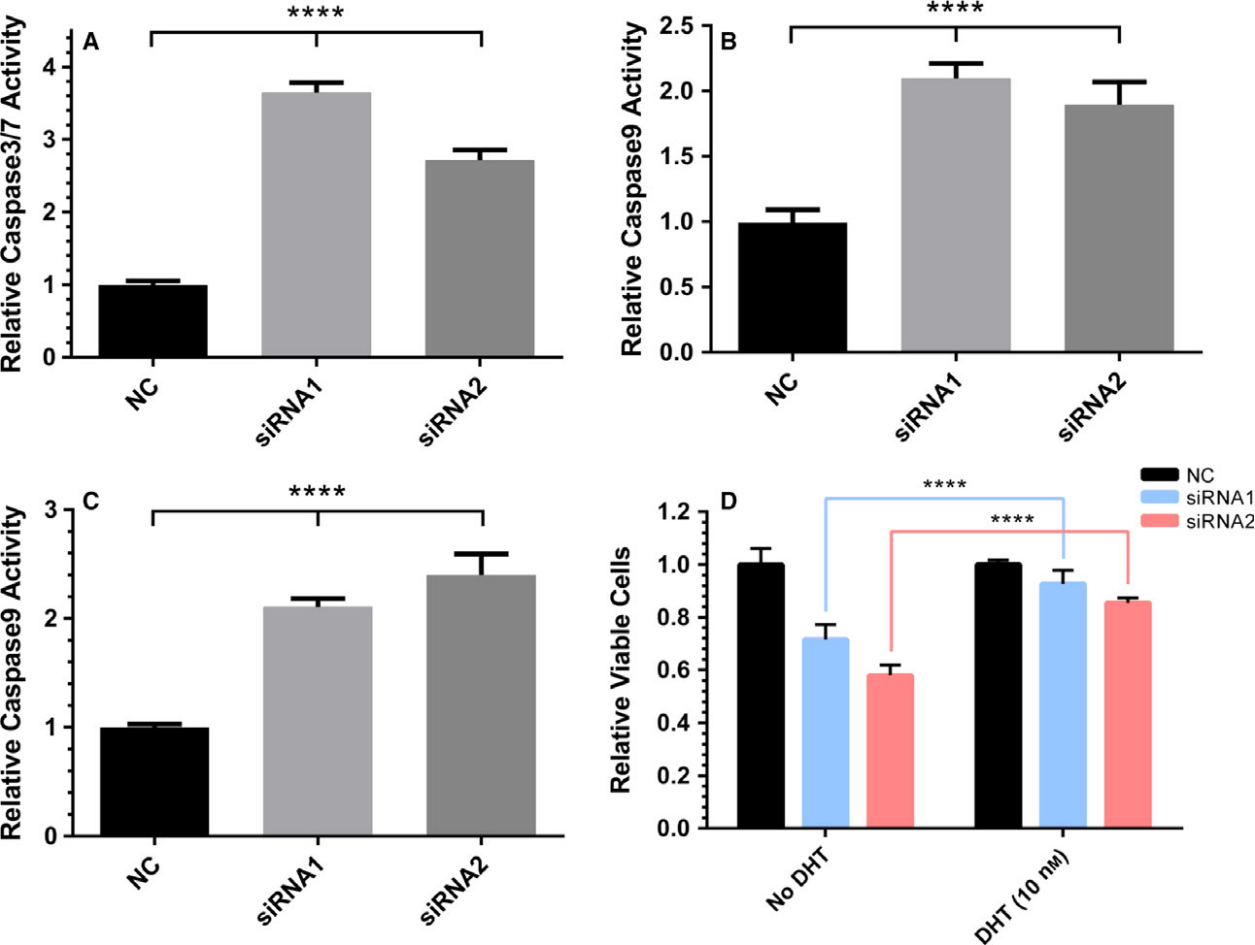
*HORAS5* knockdown induces caspase‐mediated apoptosis *in vitro*. (A–C) Caspase 3/7 (A), Caspase 8 (B), and Caspase 9 (C) activities normalized to relative LNCaP cell number 3 days post‐transfection of *HORAS5*‐siRNA vs nontargeting control siRNA. One‐way ANOVA with Tukey's posttest, ****P < 0.0001. Results are expressed as means ± SD from two independent replicates. (D) Relative viable cell counts of LNCaP cells upon knockdown of *HORAS5* (48 h) with or without DHT supplementation. Results are expressed as means ± SD from two independent replicates. Student's *t*‐test performed for statistical comparisons with ****P < 0.0001.

So far, we have shown that among the differentially expressed lincRNA, *HORAS5* is the most consistently up‐regulated gene in hormone therapy‐resistant PCa and is predominantly retained in the cell cytoplasm. To investigate the biological function of *HORAS5,* we used two distinct siRNA to knockdown this gene in LNCaP and C4‐2 cells, both capable of growing under castrate levels of testosterone, and both expressing a mutated AR gene (Sedelaar and Isaacs, 2009; Walker *et al*., 2014). With both the siRNA duplexes, we were able to achieve a robust and consistent knockdown with > 80% efficacy at a dosage as low as 2 nm (Figs 3A,B and S4). Notably, *HORAS5* knockdown significantly attenuated the growth of both LNCaP and C4‐2 cells grown in castrate levels of testosterone (Fig. 3C,D, respectively). Consistently, cell cycle analyses revealed a modest, yet significant, decrease in the fraction of cells entering the S‐phase after *HORAS5* knockdown relative to control siRNA treatment, with a parallel increase in the G0/G1 fraction (Fig. S5A,B). In accordance with the previous, induction of apoptotic caspase enzymes was observed upon treatment with both anti‐*HORAS5* siRNA (Fig. 4A–C). However, we did not observe a marked attenuation in invasion or migration potential upon siRNA‐mediated knockdown of HORAS5 at nontoxic doses (Figs S6 and S7). Together, these data strongly implicate *HORAS5* in the survival and proliferation of CRPC‐derived PCa cells.

Since *HORAS5* was up‐regulated in CRPC tumors, we questioned if its biological relevance in PCa was restricted only to castrate conditions. LNCaP cells represent an ideal model to test this hypothesis because they were derived from a castration‐resistant PCa, but physiologic levels of testosterone further stimulate their proliferation (Alisky *et al*., 2010). To this end, we knocked down *HORAS5* in LNCaP cells either in the absence or presence of DHT, a potent physiological AR agonist. Addition of DHT markedly rescued cell death observed upon *HORAS5* knockdown using both siRNA (Fig. 4D). This indicates that *HORAS5* plays a pro‐survival role primarily under castrate levels of androgen, suggesting there may be a functional association of *HORAS5* with the AR pathway.

### 3.3 Mechanisms of *HORAS5*‐dependent PCa cell survival

Our results so far indicate that *HORAS5* plays a key role in CRPC survival and growth. We next set out to investigate the molecular correlates of *HORAS5*‐dependent PCa progression. The majority of the functional studies to date have revealed that lncRNA fold into three‐dimensional structures and interact with a wide range of protein partners, thereby regulating their function (Cao *et al*., 2015). Thus, to explore the mechanisms underlying the oncogenic functions of *HORAS5,* we adopted an unbiased proteomics approach (Fig. S8).

We first knocked down *HORAS5* in LNCaP cells using two distinct siRNA and a control nontargeting siRNA. We then performed quantitative, tandem mass spectrometry proteomic analyses using treated cell lysates. Differential protein abundance analysis revealed 224 proteins that were significantly increased and 34 that were significantly decreased by greater than threefold in *HORAS5*‐knockdown cells relative to the control (Tables S3 and S4, respectively). Next, we conducted an Oncomine‐based pathway analysis of the genes that were significantly down‐regulated upon *HORAS5* silencing. The most significantly associated concept was ‘RNA polymerase II‐transcription factor activity’ (Table S5). Since AR‐RNA Polymerase II complexes have been identified as the main drivers of PCa (Wang *et al*., 2007), we decided to investigate whether the antiproliferative effect of *HORAS5* silencing was at least in part mediated by inhibition of the AR transcriptional program.

**Figure 5:**
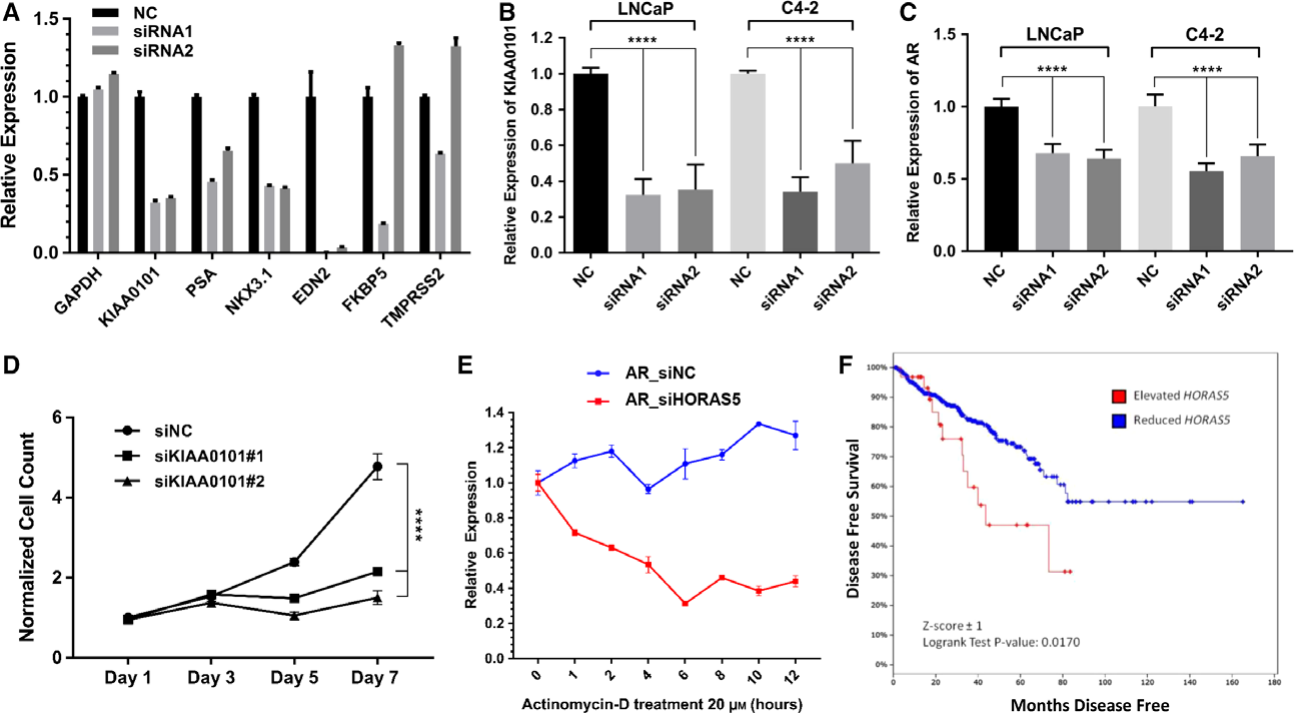
*HORAS5* regulates the expression of AR target genes and has prognostic value in PCa. (A) mRNA expression of the labeled genes after siRNA‐mediated knockdown of *HORAS5* in LNCaP cells. (B) Relative mRNA expression (qPCR) of *KIAA0101* post‐*HORAS5* knockdown in LNCaP and C4‐2 cells. (C) Relative mRNA expression (qPCR) of *AR* post‐*HORAS5* knockdown in LNCaP and C4‐2 cells. (D) MTS cell proliferation assay in C4‐2 cells upon treatment with anti‐*KIAA0101* siRNA (2 nm dose) or a control siRNA (5 nm dose). (E) Relative expression (qPCR) of *AR* mRNA in LNCaP treated with either *HORAS5*‐targeting or control siRNA (5 nm for 24 h), followed by treatment with the transcriptional inhibitor Actinomycin‐D (20 μm) for increasing durations. (F) postprostatectomy disease‐free survival based on *HORAS5* expression [cBioportal, TCGA PCa database, 456 vs 35 (Elevated *HORAS5*) patients]. All qPCR results shown as data from at least two independent replicates, statistical analyses using one‐way ANOVA with Tukey's post‐test, ****P < 0.0001.

Strikingly, upon *HORAS5* knockdown, several canonical AR targets were markedly down‐regulated (> 50%, Fig. 5A), including PSA (aka KLK3) and NKX3.1. Beyond canonical targets, a literature search revealed that several other genes that were down‐regulated upon *HORAS5* knockdown were also activated by AR. For instance, the second most down‐regulated protein, KIAA0101 (> 5‐fold), was recently confirmed as an AR‐activated gene in a study using patient specimens (Shaw *et al*., 2016). *KIAA0101* has been described as a potent oncogene in many cancers (Hosokawa *et al*., 2007; Zhu *et al*., 2013) and has been categorized as an anaphase promoting protein (Emanuele *et al*., 2011). No study to date has described the biological functions of *KIAA0101* in PCa. Thus, we set out to further explore any cellular interplay between *HORAS5* and *KIAA0101*. In CRPC C4‐2 cells, *HORAS5* knockdown led to a significant reduction in the *KIAA0101* mRNA levels by > 60% (Fig. 5B). This reduction in *KIAA0101* mRNA translated to a significant reduction of KIAA0101 protein (Fig. S9D), also verifying our initial proteomics analyses. To explore this further, we knocked down *KIAA0101* in CRPC‐derived C4‐2 cells using two distinct siRNA (Fig. S9A,B). *KIAA0101* silencing alone significantly inhibited the growth of C4‐2 cells (Fig. 5D), suggesting that the antiproliferative effect of *HORAS5* inhibition can be partly explained by downstream reduction in oncogenic AR targets like *KIAA0101*. Our proteomic data were further validated by qPCR analysis of another down‐regulated gene in *HORAS5* silenced samples, called stathmin (*STMN1*; Fig. S10A). Notably, *STMN1* is also an AR‐regulated gene (Yan *et al*., 2013) and has been previously associated with oncogenic features (Hemdan *et al*., 2014; Nie *et al*., 2015). Thus, we found several AR target genes to be markedly down‐regulated upon *HORAS5* knockdown, which prompted us to query the expression of AR itself. Remarkably, *HORAS5* knockdown significantly reduced AR expression at the transcript and protein level in both LNCaP and C4‐2 cells (Figs 5C and S10B); however, *HORAS5* itself was not regulated by AR activity (Fig. S10C). From a mechanistic standpoint, we assessed the stability of the *AR* mRNA in presence and absence of *HORAS5*. Here, we found that *HORAS5* knockdown reduced the stability of the *AR* mRNA (Fig. 5E), but found no change in the stability of the control *MYC* and *HPRT1* mRNA (Fig. S11A,B). This strongly suggests that *HORAS5* may be involved in the post‐transcriptional regulation of the *AR*. Consistently, upon *HORAS5* knockdown we found a significant decrease in the mature *AR* mRNA, but found no change in the abundance of the premature, intron‐containing *AR* transcript (Fig. S11C). Together, this evidence, and the experimental data shown in Fig. 4D, position *HORAS5* as an androgen‐independent transcript that is required for activation of the oncogenic AR transcriptional program, through post‐transcriptional maintenance of *AR* mRNA stability.

Next, to clinically interpret the *HORAS5*‐associated protein signature, we individually uploaded the differential expressed genes from the proteomics analyses into the Oncomine database. Proteins that were markedly reduced in response to *HORAS5* knockdown strongly correlated with increased odds of disease recurrence at 3 and 5 years after primary treatment (Fig S8). Direct evaluation of *HORAS5* expression in PCa patient samples also confirmed that higher expression of this lincRNA predicted poorer clinical outcome in terms of overall survival (Fig. 5F). These clinical correlations are consistent with *HORAS5* mediating major proliferative functions in PCa under hormone‐depleted conditions as shown above. The proteins that were up‐regulated upon *HORAS5* knockdown returned no significant association (*P* < 0.001) with any clinical variables. Next, we queried the TCGA PCa transcriptomic data to identify genes that have significant positive correlation with *HORAS5* expression. Using these genes, we then performed a computational analyses, called BART (Amaral *et al*., 2018), to predict the common regulatory transcription factors. Consistent with the involvement of *HORAS5* in maintenance of AR‐signaling, BART analyses predicted AR to be one of top regulatory factors for the *HORAS5*‐associated genes in patient tumors (Fig. S11D). As expected, similar analyses for negatively correlated genes did not return the AR as a significant transcription factor (Fig. S11D).

### 3.4 Therapeutic potential of *HORAS5* inactivation

**Figure 6:**
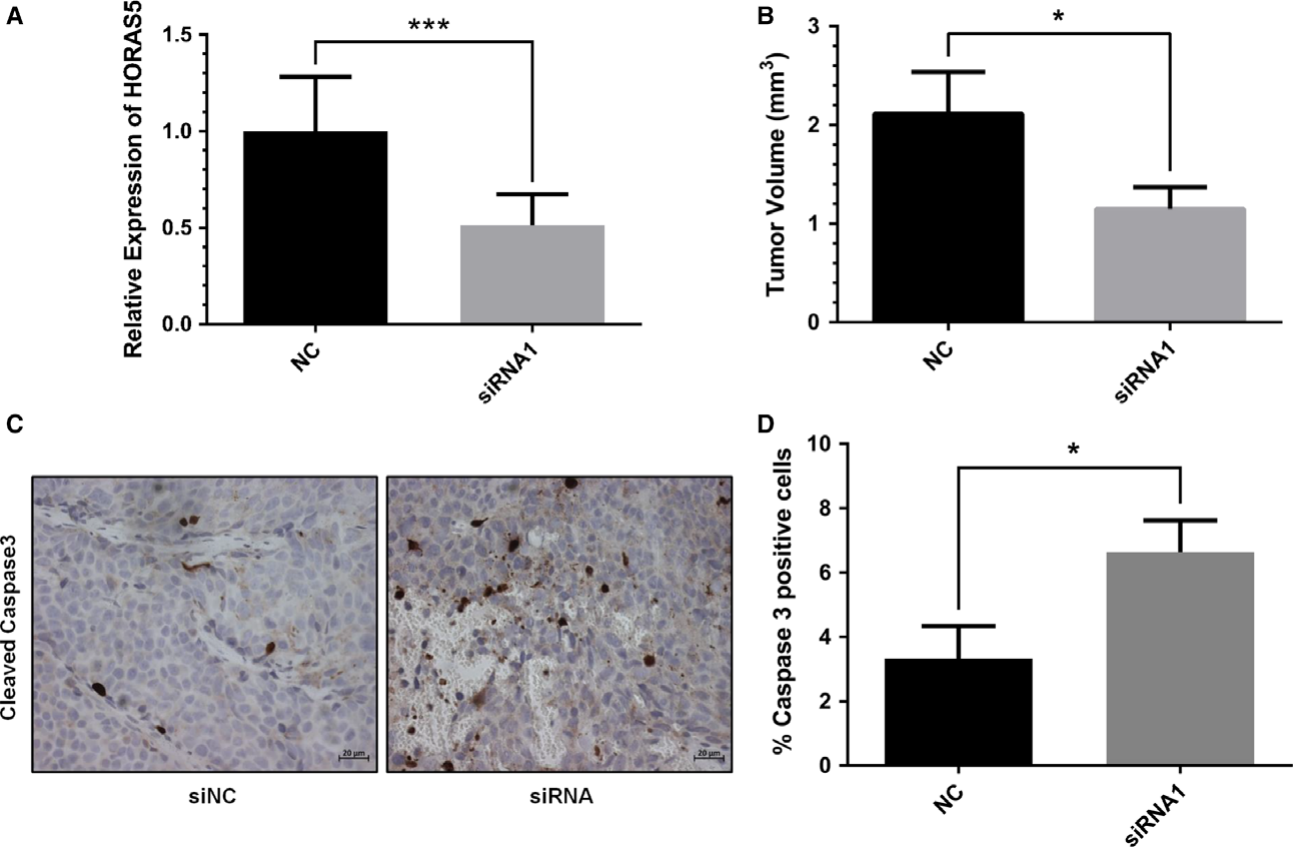
*HORAS5* knockdown attenuates tumor growth *in vivo*. (A) Relative expression of *HORAS5* (qPCR) 72 h following AteloGene:siRNA injections (*n* = 3 in each arm). Raw values were normalized to *GAPDH* and *HPRT1* and shown relative to the control group (***P < 0.001). (B) Quantitative measurements of tumor volume postknockdown of *HORAS5* (72 h). Statistical significance from Student's *t*‐test with *P < 0.05. (C) Representative IHC images for cleaved Caspase 3 from control siRNA (NC) and anti‐*HORAS5* siRNA treatment groups. The scale bar at the bottom marks a 20 μm width. (D) Overall IHC staining quantification from C. Results are expressed as percent of positive cells to total cells in the visual field. Data are shown as means ± SD, and a Student's *t*‐test was used to calculate significance, *P < 0.05.

Thus far, we have established that *HORAS5* drives CRPC growth by supporting oncogenic AR transcriptional programs and cellular availability of several oncogenic proteins. We then sought to validate the oncogenic roles of *HORAS5 in vivo*. For this, we generated *in vivo* androgen‐insensitive sublines of LNCaP as previously described (Kuruma *et al*., 2013). Using the AteloGene delivery system (Takeshita *et al*., 2005), after 2 consecutive siRNA injections, we were able to achieve a significant knockdown of *HORAS5* by about 50% (Fig. 6A). This inhibition of *HORAS5* expression ensued in a significant reduction in tumor volume relative to the control (Fig. 6B). To confirm whether this reduction was due to apoptotic killing of tumor cells, we generated tissue microarrays from the treated tumors and stained for active/cleaved Caspase 3. Complementing the reduction in tumor volume, inhibition of *HORAS5* led to significantly higher staining for cleaved Caspase 3 in tumor cells (Fig. 6C,D). These results indicate that *HORAS5* modulates CRPC cell proliferation and survival *in vivo* and that it could be used as a novel therapeutic target for CRPC.

## 4 Discussion

In this study, we have identified *HORAS5* as a mediator of CRPC cell proliferation and as a potential therapeutic and prognostic target for PCa. Preclinical data obtained using different cell lines indicate that *HORAS5* is required for CRPC cell survival in castrate levels of testosterone. In keeping with this hypothesis, we showed that *HORAS5* is highly expressed in early clinical PCa specimens and in CRPC PDX. Additionally, higher *HORAS5* expression in primary PCa samples predicts poorer clinical outcome.

Our results also indicate that *HORAS5* is predominantly contained in the cytoplasm of PCa cells where it regulates the stability of the *AR* mRNA. In osteosarcoma cells, this lncRNA has been recently shown to be associated with the AGO complex, where it buffers miRNA 645 (mir‐645) and indirectly increases the levels of the pro‐apoptotic interferon‐induced protein with tetratricopeptide repeats two protein (Wang *et al*., 2016b). However, our proteomic (Tables S3 and S4) and miRNA binding prediction data (Fig. S12A) did not show the existence of this pathway in PCa cells, where the lncRNA seems to play an oncogenic role. Moreover, mir‐645 was not expressed in AR‐positive PCa cells (Fig. S12B). Instead, our results are more in line with recent evidence showing that *HORAS5* overexpression is associated with worse prognosis in hepatocellular carcinoma patients (Xu *et al*., 2017). The previous study also showed that *HORAS5* drives the metastatic potential of liver cancer cells. Taken together, this evidence indicates that the effects of *HORAS5* expression are tissue‐ and disease‐specific, as described for other lncRNA (Huarte, 2015; Schmitt and Chang, 2016).

In this study, we have shown using different techniques that *HORAS5* silencing leads to a marked inhibition of the *KIAA0101* oncogene. *KIAA0101* has been described as a pro‐survival oncogene in several cancer types (Hosokawa *et al*., 2007; Zhu *et al*., 2013) and as a driver of cell cycle progression (Emanuele *et al*., 2011). Our data show that *HORAS5* silencing attenuates cell cycle progression and induces caspase‐dependent apoptosis in CRPC‐derived cells. Interestingly, *HORAS5* silencing significantly attenuates the AR transcriptional program, with marked reduction in oncogenic AR targets such as *KIAA0101*. This can be explained by the decrease in the expression of *AR* mRNA itself likely due to reduced cytoplasmic stability in absence of *HORAS5*. Several other oncogenic proteins are positively associated with *HORAS5* expression as well (Table S3). This suggests that targeting *HORAS5* could simultaneously block multiple key oncogenic pathways in CRPCs.

Other lncRNA have been previously identified as potential drivers of CRPC. In particular, uncharacterized long noncoding RNA (*LOC283070*) has been shown to induce the proliferation of LNCaP cells in androgen‐depleted media (Wang *et al*., 2016a). Since this lncRNA had not been described before, we decided to analyze its expression in our models. Unfortunately, we were unable to do so, since we found that the primers used to measure *LOC283070* expression perfectly overlap with the CAMK1D protein, which has been identified as a potential interactor of *LOC283070*. In addition, the siRNA sequences used to silence *LOC283070* show a substantial overlap with the CAMK1D protein. Based on these findings, we question the assumption that *LOC283070* is a true lncRNA. On the contrary, homeobox transcript antisense RNA (*HOTAIR*) is a well‐characterized oncogenic lncRNA, which is mainly expressed in the nucleus and binds directly to the AR. A recent study identified HOTAIR as a putative driver of CRPC progression (Zhang *et al*., 2015). Notably, this study did not test the therapeutic potential of *HOTAIR* knockdown *in vivo*. Hence, this manuscript reports the functional characterization of a lncRNA that drives CRPC *in vitro* and *in vivo*.

## 5 Conclusion

Our study indicates that the cytoplasmic lncRNA, *HORAS5*, mediates CRPC progression in an AR‐dependent manner by regulating AR mRNA stability, and is associated with poorer clinical outcome in human PCa samples. In addition, our *in vivo* data further implicate lncRNA as putative therapeutic targets for CRPC (Fig. 6). Thus, our study adds to the mounting evidence that centrally implicates lncRNA in cancer biology, and warrants the development of innovative therapies that target oncogenic noncoding RNA molecules in advanced, lethal PCa.

## Conflict of interest

The authors declare no conflict of interest.

## Author contributions

AP and EV conducted most of the experiments and wrote the manuscript. HX, DL, and RW conducted the *in vivo* experiments. RM and PP conducted *in silico* analyses. JR conducted proteomics analyses. JRE and FF analyzed RNA sequencing data. CCC critically revised the paper. YW and FC ideated and supervised the study, and critically revised the paper.

## Supporting information


**Fig. S1.** Tumor volume and serum PSA levels for LTL313B/BR paired PDX‐pair in NOD/SCID mice.

**Fig. S2.** Basal *HORAS5* expression in a panel of normal and cancerous tissues.

**Fig. S3.** Expression of Long and short *HORAS5* splice variants in PCa cell lines and LTL PDXs.

**Fig. S4.** *HORAS5* short transcript knockdown in two PCa cell lines.

**Fig. S5.** *HORAS5* knockdown affects cell cycle progression *in vitro*.

**Fig. S6.** *HORAS5* silencing does not alter cell migration potential.

**Fig. S7.** *HORAS5* silencing does not mediate cellular invasion.

**Fig. S8.** Proteins down‐regulated in response to *HORAS5* knockdown are associated with clinical PCa recurrence.

**Fig. S9.** *KIAA0101* knockdown in CRPC‐derived PCa cells.

**Fig. S10.** Several key oncogenic proteins are down‐regulated upon *HORAS5* knockdown.

**Fig. S11.** Functional regulation of AR activity by *HORAS5*.

**Fig. S12**. *HORAS5‐*miRNA interaction predictions.

**Table S1.** LincRNAs up‐regulated in LTL‐313BR (hormone‐independent) vs LTL‐313B (hormone‐dependent).

**Table S2.** LincRNAs down‐regulated in LTL‐313BR (hormone‐independent) vs LTL‐313B (hormone‐dependent).

**Table S3.** List of proteins that are up‐regulated (FC ≥ 3) upon HORAS5 knockdown in LNCaP cells.

**Table S4.** List of proteins that are down‐regulated (FC≤3) upon HORAS5 knockdown in LNCaP cells.

**Table S5**. Top biological concepts significantly associated with genes downregulated upon HORAS5 knockdown.

**Table S6.** List of pre‐designed Taqman probes used for qPCR analyses.
