# Corrigendum to: The long noncoding RNA HORAS5 mediates castration‐resistant prostate cancer survival by activating the androgen receptor transcriptional program

**DOI:** 10.1002/1878-0261.12618

**Published:** 2020-02-04

In the Abstract and Keywords of their paper, Parolia et al. (2019) omitted to indicate that the long noncoding RNA *HORAS5 *is formally known as *Linc00161.* The authors had provisionally named *Linc00161* as *HORAS5* to illustrate its role in hormone‐resistant prostate cancer. The amended Abstract and Keywords, stating the formal name of HORAS5, are included below.

## Abstract

Prostate cancer (PCa) is driven by the androgen receptor (AR) signaling axis. Hormonal therapy often mitigates PCa progression, but a notable number of cases progress to castration‐resistant PCa (CRPC). CRPC retains AR activity and is incurable. Long noncoding RNA (lncRNA) represent an uncharted region of the transcriptome. Several lncRNA have been recently described to mediate oncogenic functions, suggesting that these molecules can be potential therapeutic targets. Here, we identified CRPC‐associated lncRNA by analyzing patient‐derived xenografts (PDXs) and clinical data. Subsequently, we characterized one of the CRPC‐promoting lncRNA,* HORAS5* **(also known as Linc00161),** *in vitro* and *in vivo*. We demonstrated that *HORAS5* is a stable, cytoplasmic lncRNA that promotes CRPC proliferation and survival by maintaining AR activity under androgen‐depleted conditions. Most strikingly, knockdown of *HORAS5* causes a significant reduction in the expression of AR itself and oncogenic AR targets such as KIAA0101. Elevated expression of *HORAS5* is also associated with worse clinical outcomes in patients. Our results from *HORAS5* inhibition in *in vivo* models further confirm that *HORAS5* is a viable therapeutic target for CRPC. Thus, we posit that *HORAS5* is a novel, targetable mediator of CRPC through its essential role in the maintenance of oncogenic AR activity. Overall, this study adds to our mechanistic understanding of how lncRNA function in cancer progression.

## Keywords

androgen independence; HORAS; HORAS5; **Linc00161**; lncRNA; prostate cancer
