# Supplementary material for: The long noncoding RNA HORAS5 mediates castration‐resistant prostate cancer survival by activating the androgen receptor transcriptional program
Source: Mol Oncol. 2019 Mar 5;13(5):1121–36. doi: 10.1002/1878-0261.12471 (PMC6487714; doi:10.1002/1878-0261.12471)
Supplement: Supplementary file 1 — Fig. S1. Tumor volume and serum PSA levels for LTL313B/BR paired PDX‐pair in NOD/SCID mice. [file MOL2-13-1121-s001.pdf]

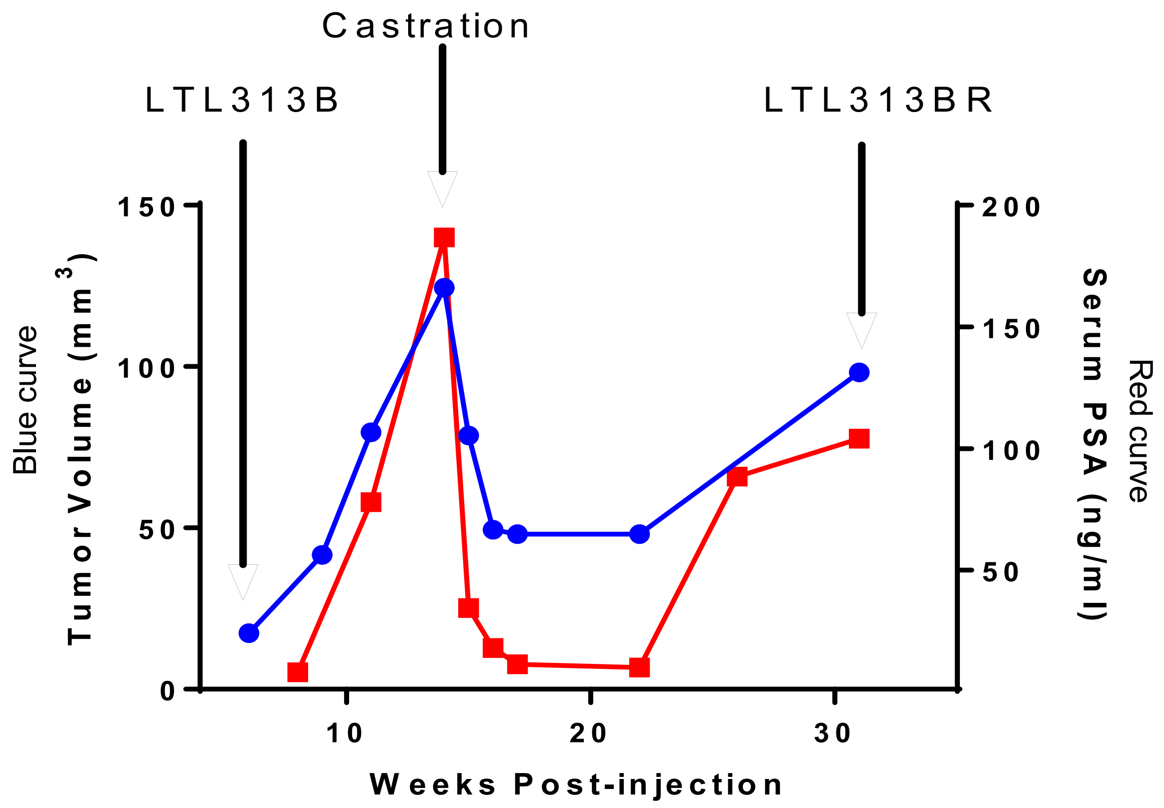

Supplementary Figure 1 | Tumor volume and serum PSA levels for LTL313B/BR paired PDX-pair in NOD/SCID mice. Tumor volume (blue line) and serum PSA (red line) dramatically increase after LTL313B tumor engraftment. When tumors reach ~150mm3 volume, mice were surgically castrated. In response to castration, tumor volume and PSA both sharply decline. However, tumor eventually relapses around week 20 with concurrent increase in serum PSA levels. The castration-resistant tumor is referred to as LTL313BR.
