# Supplementary material for: The long noncoding RNA HORAS5 mediates castration‐resistant prostate cancer survival by activating the androgen receptor transcriptional program
Source: Mol Oncol. 2019 Mar 5;13(5):1121–36. doi: 10.1002/1878-0261.12471 (PMC6487714; doi:10.1002/1878-0261.12471)
Supplement: Supplementary file 2 — Fig. S2. Basal HORAS5 expression in a panel of normal and cancerous tissues. [file MOL2-13-1121-s002.pdf]

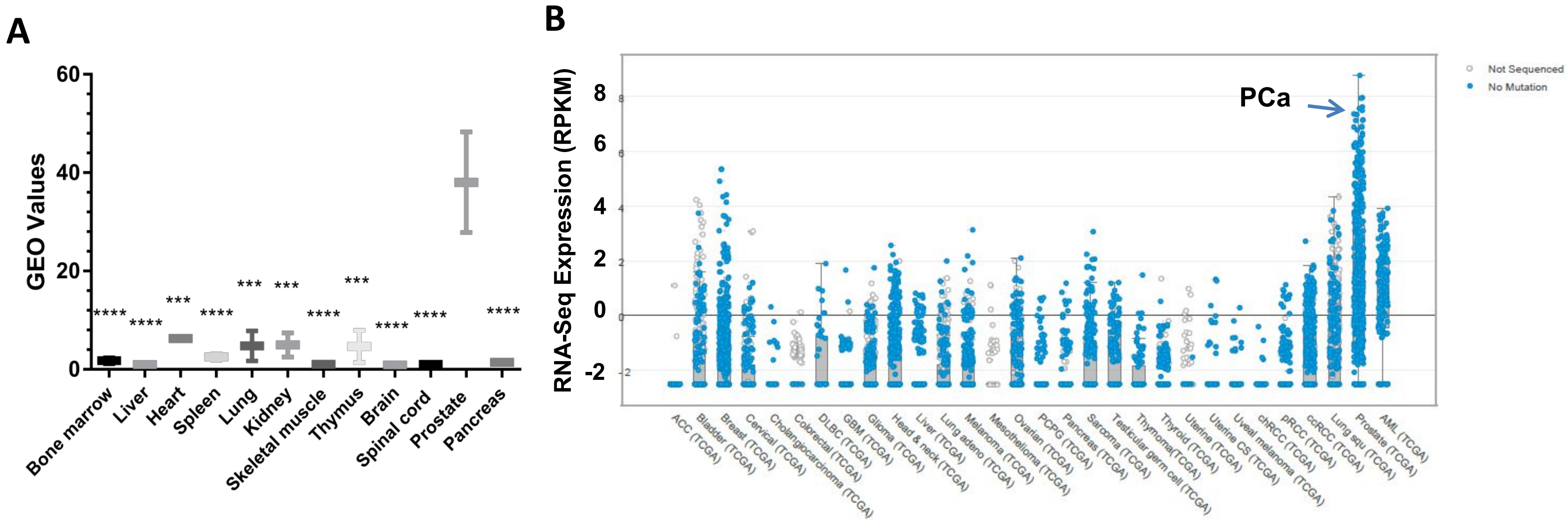

Supplementary Figure 2 | Basal *HORAS5* expression in a panel of normal and cancerous tissues. (A) *HORAS5* expression is highest in healthy prostate tissue relative to 11 other tissue types. This microarray data was downloaded from GEO Profiles (ID: 2921923) and replotted using GraphPad. **(B)** CBioPortal analysis of *HORAS5* abundance in multiple cancerous tissues.
