# Supplementary material for: The long noncoding RNA HORAS5 mediates castration‐resistant prostate cancer survival by activating the androgen receptor transcriptional program
Source: Mol Oncol. 2019 Mar 5;13(5):1121–36. doi: 10.1002/1878-0261.12471 (PMC6487714; doi:10.1002/1878-0261.12471)
Supplement: Supplementary file 3 — Fig. S3. Expression of Long and short HORAS5 splice variants in PCa cell lines and LTL PDXs. [file MOL2-13-1121-s003.pdf]

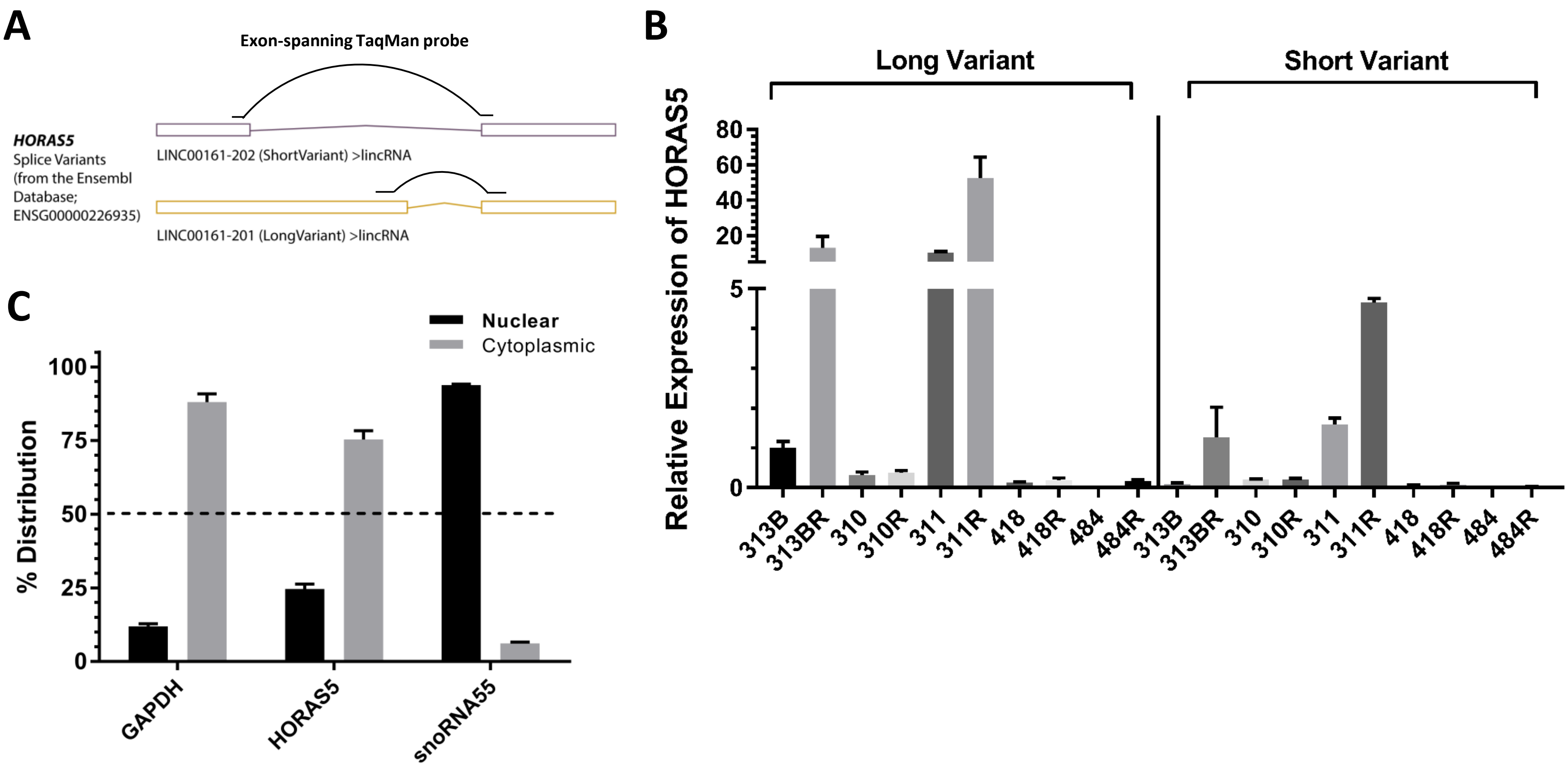

Supplementary Figure 3: Expression of Long and short *HORAS5* splice variants in PCa cell lines and LTL PDXs. (A) Schematic showing the two *HORAS5* splice variants and the exon-spanning TaqMan probes used for qPCR to distinguish the two variants. **(B)** Basal expression (TaqMan qPCR) of both *HORAS5* transcript variants in 5 different paired hormone-sensitive/-resistant LTL PDXs. Data is plotted as mean ± S.D. from a representative sample and is normalized to the *HORAS5* long transcript variant expression in the 313B sample for clarity. **(C)** qPCR expression of *HORAS5* in the nuclear and cytoplasmic sub-cellular fractions of AR-positive C4-2 cells. *GAPDH* and *snoRNA5* are used as controls for cytoplasmic and nuclear fractions, respectively. Results from two independent experiments are plotted.
