# Supplementary material for: The long noncoding RNA HORAS5 mediates castration‐resistant prostate cancer survival by activating the androgen receptor transcriptional program
Source: Mol Oncol. 2019 Mar 5;13(5):1121–36. doi: 10.1002/1878-0261.12471 (PMC6487714; doi:10.1002/1878-0261.12471)
Supplement: Supplementary file 4 — Fig. S4. HORAS5 short transcript knockdown in two PCa cell lines. [file MOL2-13-1121-s004.pdf]

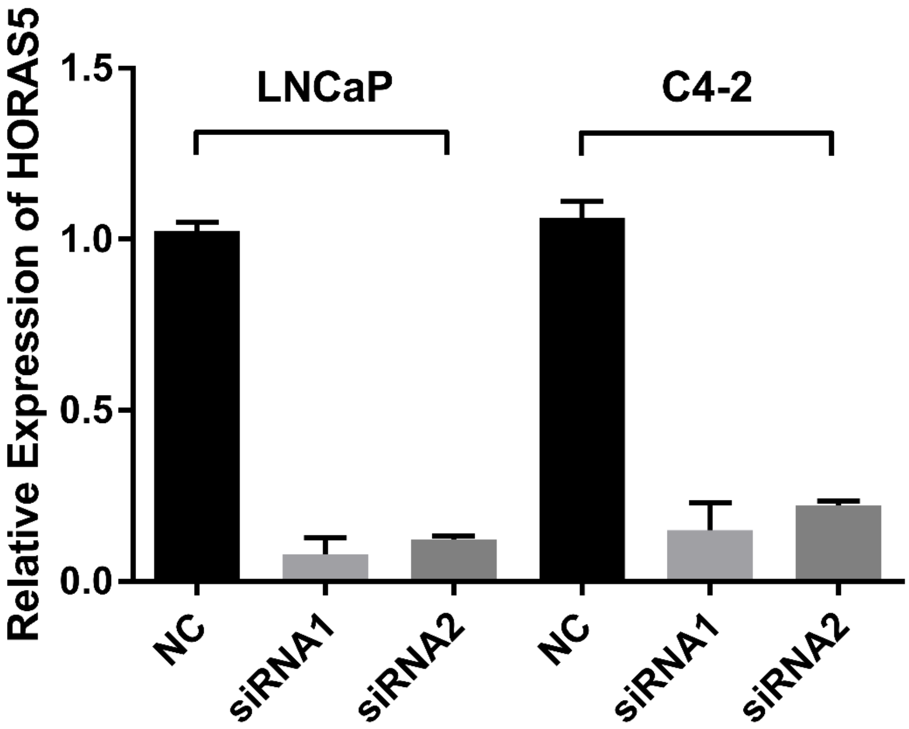

Supplementary Figure 4 | *HORAS5* short transcript knockdown in two prostate cancer cell lines. LNCaP and C4-2 cells were treated with either a NC scramble or anti-*HORAS5* siRNAs for 48hrs prior to qPCR analysis. Data is depicted as means ± S.D. from a representative experiment.
