# Supplementary material for: The long noncoding RNA HORAS5 mediates castration‐resistant prostate cancer survival by activating the androgen receptor transcriptional program
Source: Mol Oncol. 2019 Mar 5;13(5):1121–36. doi: 10.1002/1878-0261.12471 (PMC6487714; doi:10.1002/1878-0261.12471)
Supplement: Supplementary file 5 — Fig. S5. HORAS5 knockdown affects cell cycle progression in vitro. [file MOL2-13-1121-s005.pdf]

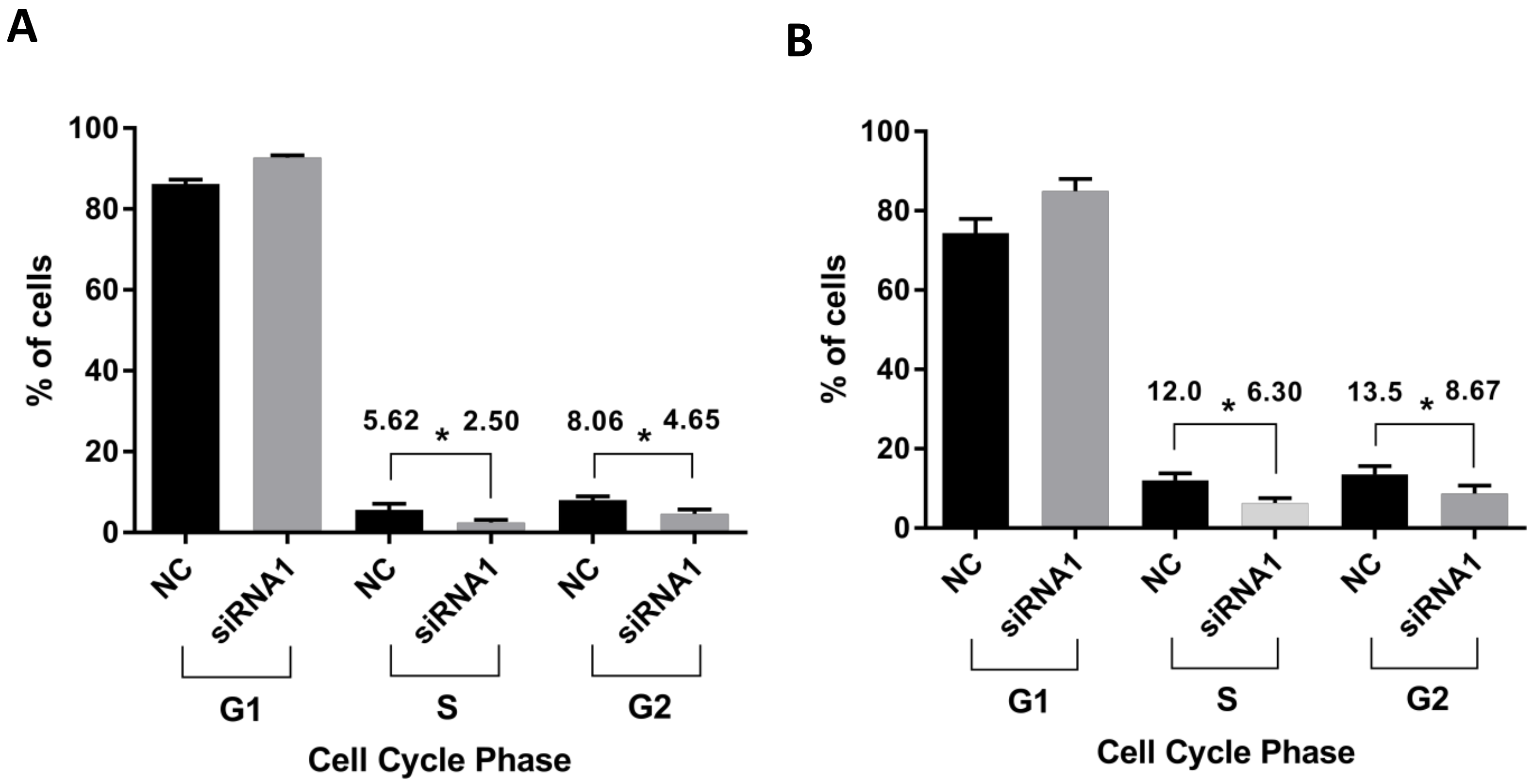

Supplementary Figure 5 | ***HORAS5* knockdown affects cell cycle progression *in vitro.*** **(A,B)** Fraction of LNCaP **(A)** and C4-2 **(B)** cells in different phases of the cell cycle 72hrs post-knockdown of *HORAS5*. Student *t*-test were used to measure significance, *P<0.05. Cell cycle data shown as means ± S.D. from three independent experiments.
