# Supplementary material for: The long noncoding RNA HORAS5 mediates castration‐resistant prostate cancer survival by activating the androgen receptor transcriptional program
Source: Mol Oncol. 2019 Mar 5;13(5):1121–36. doi: 10.1002/1878-0261.12471 (PMC6487714; doi:10.1002/1878-0261.12471)
Supplement: Supplementary file 6 — Fig. S6. HORAS5 silencing does not alter cell migration potential. [file MOL2-13-1121-s006.pdf]

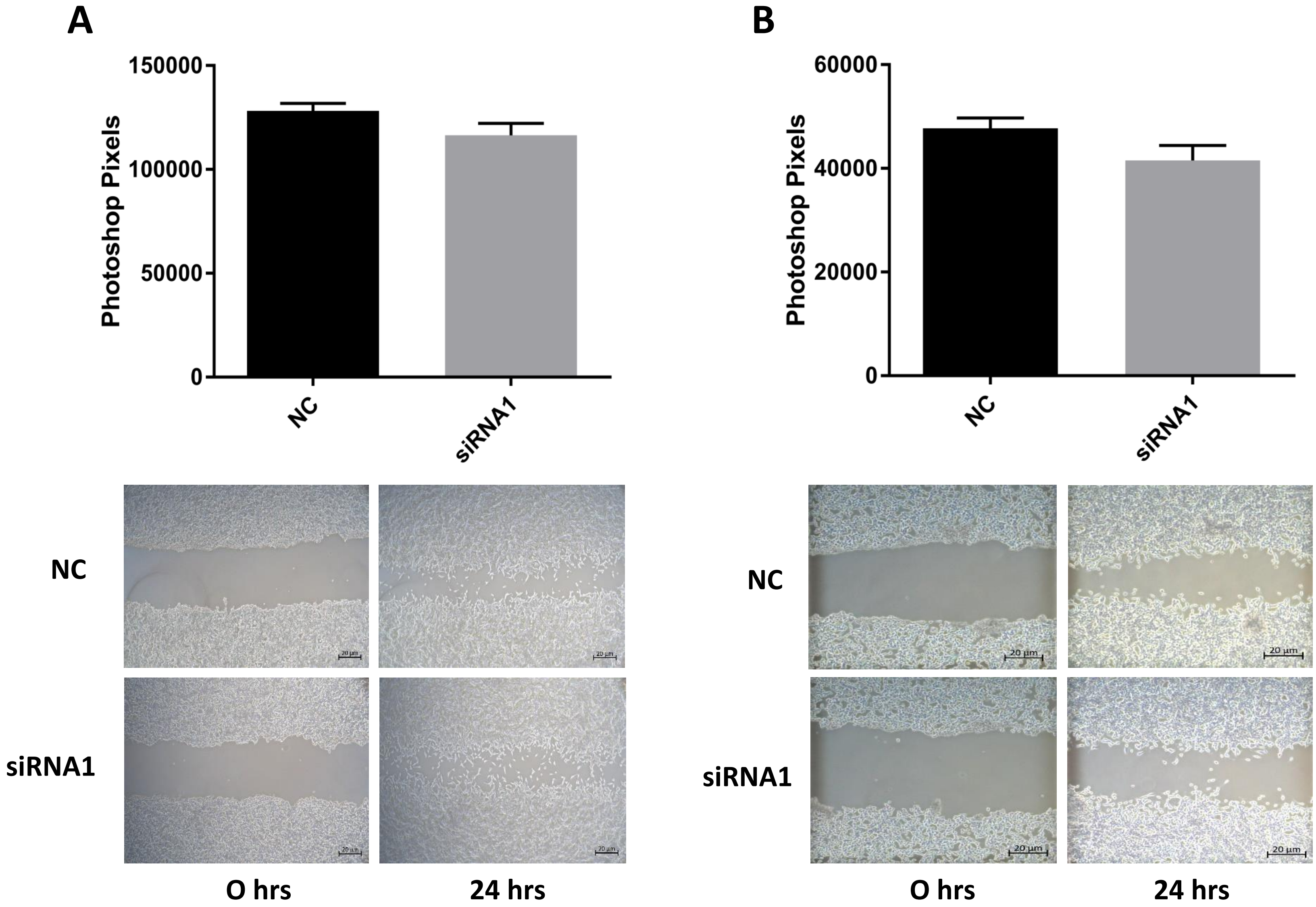

Supplementary Figure 6 | *HORAS5* silencing does not alter cell migration potential. (A,B) Wound-healing (scratch) assays following 18hr knockdown of *HORAS5* in LNCaP **(A)** and C4-2 **(B)** cells. Results are shown as means ± S.D. from three independent experiments. Significance tested using a Student's *t*-test. Counted by BioRad cell counter and plated at 2.0*10^6 cells per 6-well for LNCaP cells, and 3.5*10^6 cells per 60mm dish for C4-2 cells. Only LNCaP cells in 6-well plates required pre-coating with poly-L-lysine for 4 hours (rinsed with dH20 after coating). Cells were left in RPMI-1640 serum-free for 24 hours post -scratch prior to image capture with AxioVision camera and photoshop pixel quantification. The scale bars shown the 20μm width.
