# Supplementary material for: The long noncoding RNA HORAS5 mediates castration‐resistant prostate cancer survival by activating the androgen receptor transcriptional program
Source: Mol Oncol. 2019 Mar 5;13(5):1121–36. doi: 10.1002/1878-0261.12471 (PMC6487714; doi:10.1002/1878-0261.12471)
Supplement: Supplementary file 7 — Fig. S7. HORAS5 silencing does not mediate cellular invasion. [file MOL2-13-1121-s007.pdf]

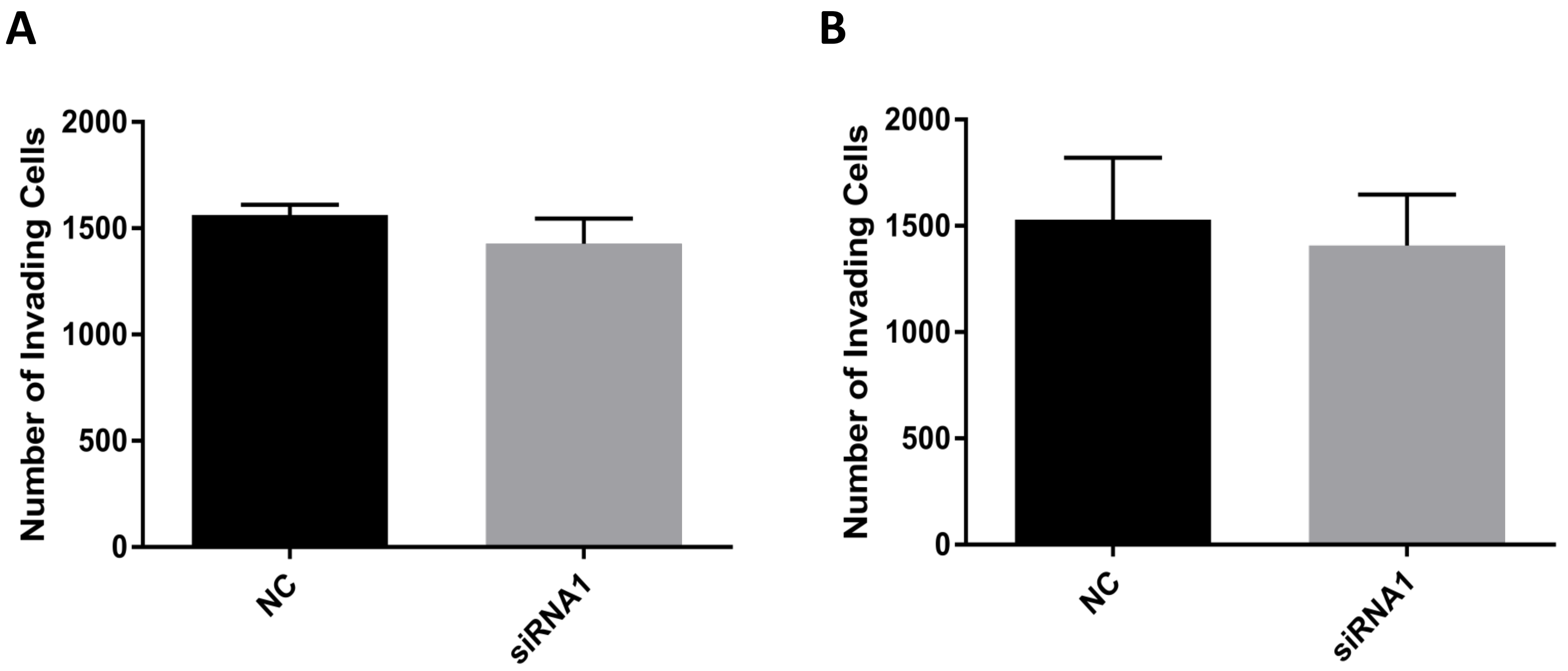


**Supplementary Figure 7 | *HORAS5* silencing does not mediate cellular invasion.** LNCaP **(A)** and C4-2 **(B)** cell invasive potential examined after 18hr knockdown of *HORAS5.* Boyden chamber 48-well invasion assays were utilized and results are shown as means ± S.D. from three independent trials. Significance tested using a Student's *t*-test.
