# Supplementary material for: The long noncoding RNA HORAS5 mediates castration‐resistant prostate cancer survival by activating the androgen receptor transcriptional program
Source: Mol Oncol. 2019 Mar 5;13(5):1121–36. doi: 10.1002/1878-0261.12471 (PMC6487714; doi:10.1002/1878-0261.12471)
Supplement: Supplementary file 8 — Fig. S8. Proteins down‐regulated in response to HORAS5 knockdown are associated with clinical PCa recurrence. [file MOL2-13-1121-s008.pdf]

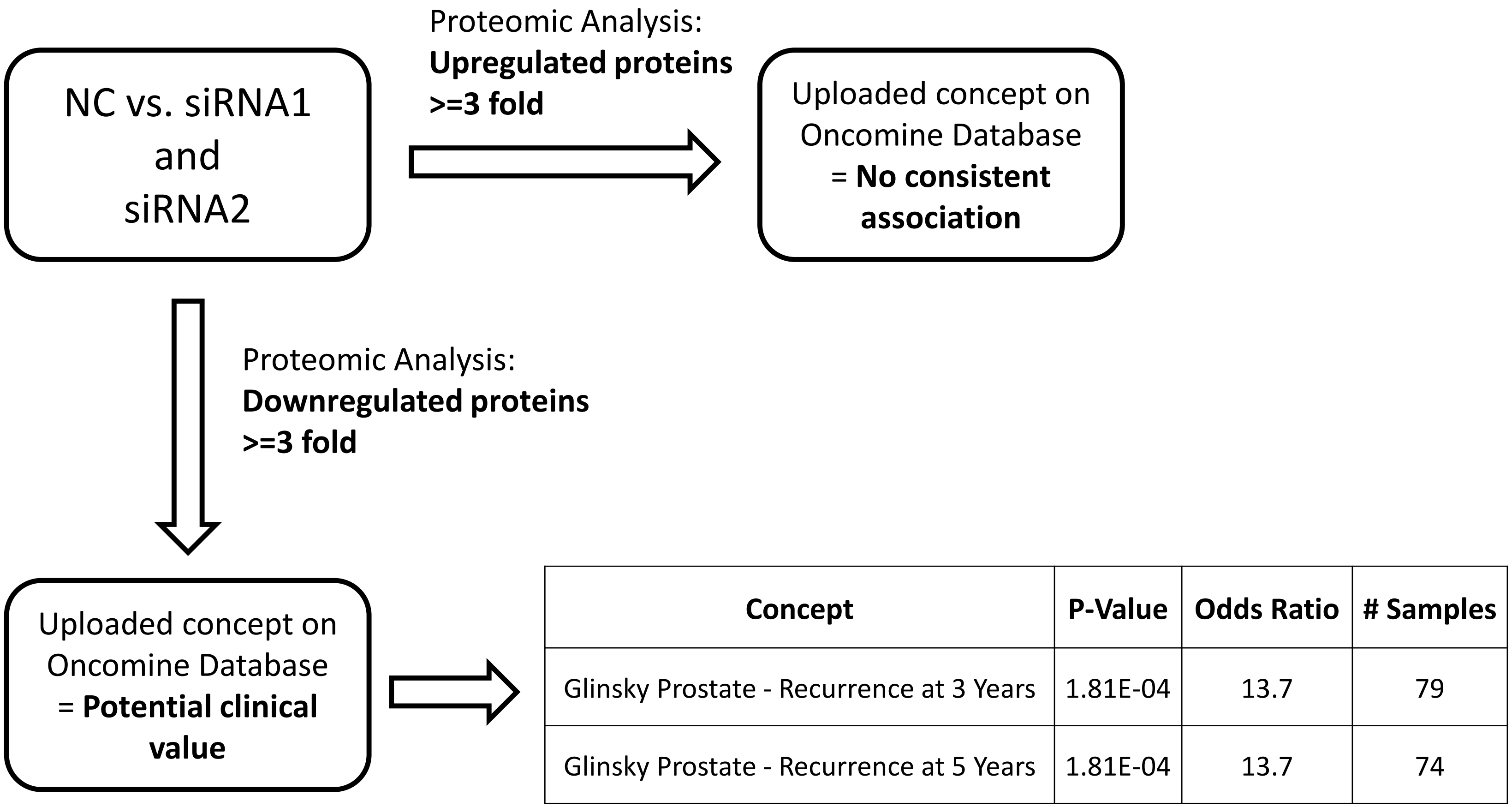

Concept	P-Value	Odds Ratio	# Samples
Glinsky Prostate - Recurrence at 3 Years	1.81E-04	13.7	79
Glinsky Prostate - Recurrence at 5 Years	1.81E-04	13.7	74

Supplementary Figure 8 | Proteins down-regulated in response to *HORAS5* knockdown are associated with clinical PCa recurrence. Differentially expressed protein genes (Supplemental tables 3 and 4) identified from *in vitro* knockdown of *HORAS5* in LNCaP cells followed by MS/MS proteomics analysis were investigated for clinical associations using the Oncomine web portal. Proteins up and down-regulated (≥ 3-fold) in LTL313BR *versus* LTL313B were uploaded into Oncomine separately, and only significant clinical associations are reported above.
