# Supplementary material for: The long noncoding RNA HORAS5 mediates castration‐resistant prostate cancer survival by activating the androgen receptor transcriptional program
Source: Mol Oncol. 2019 Mar 5;13(5):1121–36. doi: 10.1002/1878-0261.12471 (PMC6487714; doi:10.1002/1878-0261.12471)
Supplement: Supplementary file 9 — Fig. S9. KIAA0101 knockdown in CRPC‐derived PCa cells. [file MOL2-13-1121-s009.pdf]

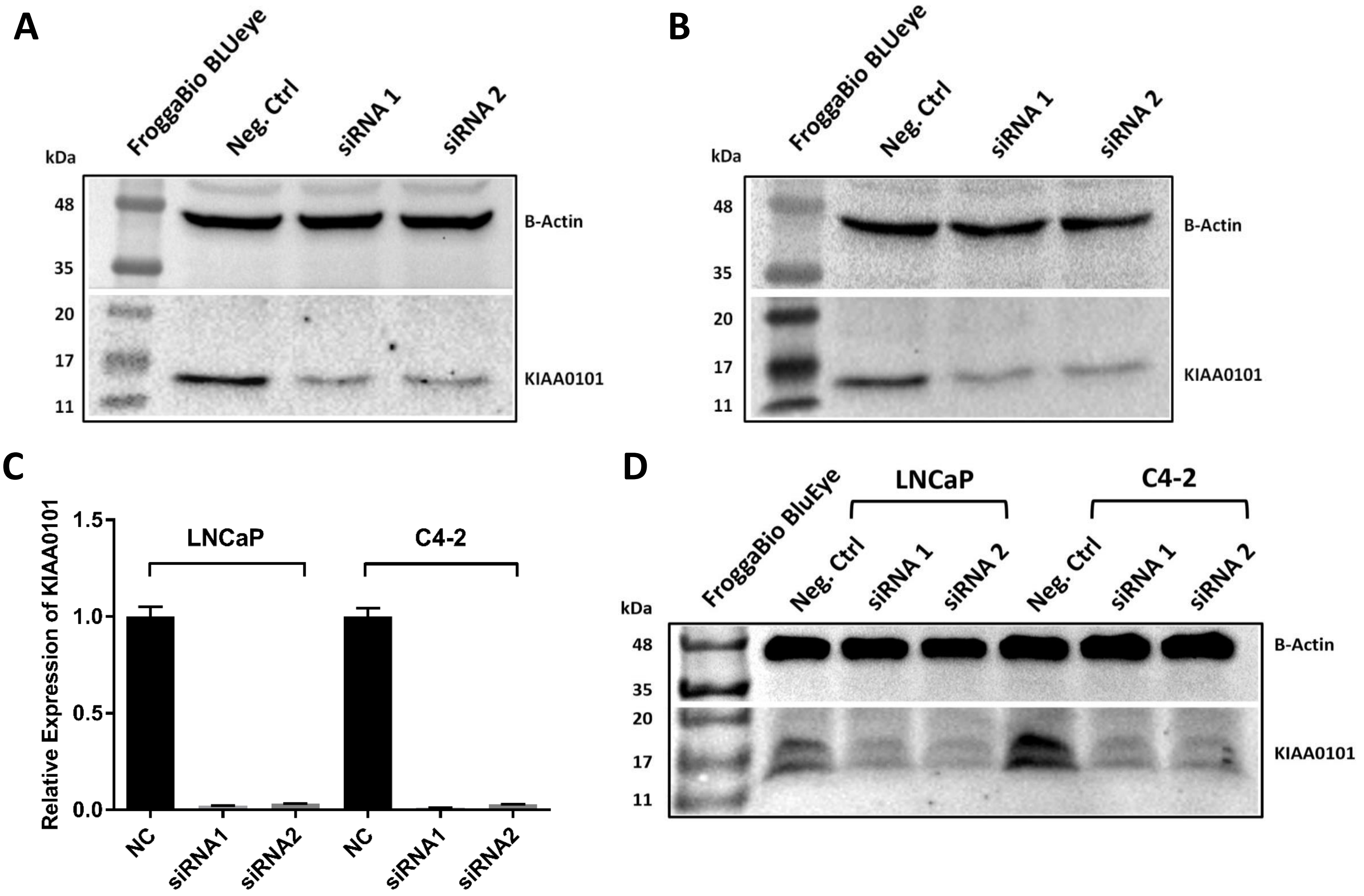

Supplementary Figure 9 | *KIAA0101* knockdown in CRPC-derived PCa cells. (A,B) Immunoblots showing B-Actin (control) and KIAA0101 protein expression 72 hours after knockdown of *HORAS5* in LNCaP **(A)** and C4-2 **(B)** cells. **(C)** Relative mRNA expression (qPCR) of *KIAA0101* post knockdown in LNCaP and C4-2 cells. Results are from a representative sample. **(D)** Immunoblot showing B-Actin (control) and KIAA0101 protein expression 72 hours after knockdown of *KIAA0101*_by control and targeted DsiRNAs in LNCaP and C4-2 cells. All blot were cut for primary antibody incubation, and have been boxed to indicate results which are from the same run.
