# Supplementary material for: The long noncoding RNA HORAS5 mediates castration‐resistant prostate cancer survival by activating the androgen receptor transcriptional program
Source: Mol Oncol. 2019 Mar 5;13(5):1121–36. doi: 10.1002/1878-0261.12471 (PMC6487714; doi:10.1002/1878-0261.12471)
Supplement: Supplementary file 10 — Fig. S10. Several key oncogenic proteins are down‐regulated upon HORAS5 knockdown. [file MOL2-13-1121-s010.pdf]

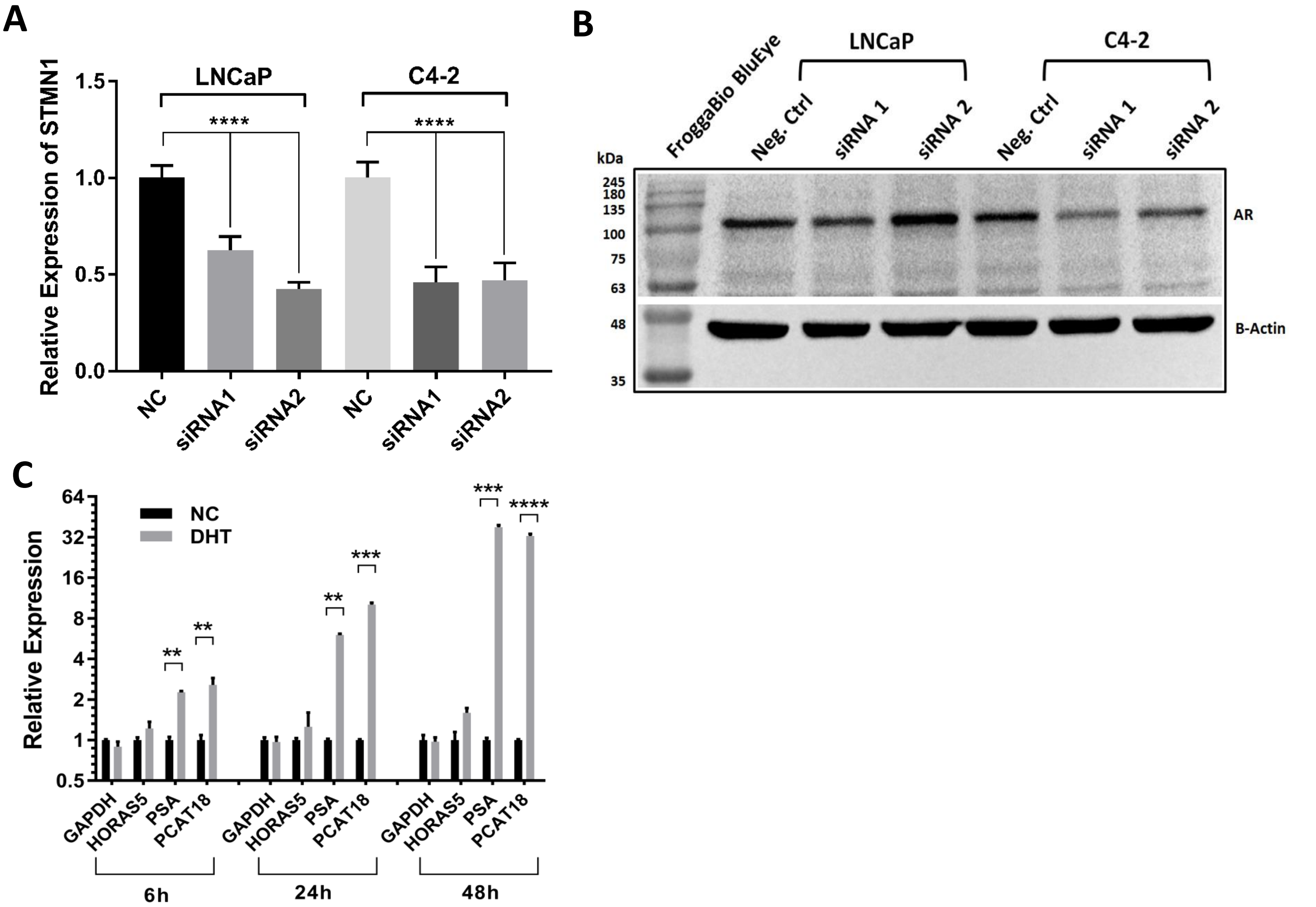

Supplementary Figure 10 | Several key oncogenic proteins are down-regulated upon *HORAS5* knockdown. (A) *STMN1* mRNA expression (qPCR) in LNCaP and C4-2 cells 72 hours post-knockdown of *HORAS5*. One-way ANOVA with Tukey's Post Test. qPCR data are shown as means of three independent replicates ± S.D.. **(B)** Immunoblot of the AR and control B-actin 72h after the knockdown of *HORAS5* using two distinct siRNAs (2nM dosage) in LNCaP and C4-2 cells. Immunoblot data shown as a representative sample. **(C)** mRNA expression of labeled genes in LNCaP cells following DHT addition (10nM) for various durations. A single representative experiment is shown here. Student's *t*-test performed for statistical comparisons with **P<0.005, ***P<0.001, and ****P<0.0001.
