# Supplementary material for: The long noncoding RNA HORAS5 mediates castration‐resistant prostate cancer survival by activating the androgen receptor transcriptional program
Source: Mol Oncol. 2019 Mar 5;13(5):1121–36. doi: 10.1002/1878-0261.12471 (PMC6487714; doi:10.1002/1878-0261.12471)
Supplement: Supplementary file 11 — Fig. S11. Functional regulation of AR activity by HORAS5. [file MOL2-13-1121-s011.pdf]

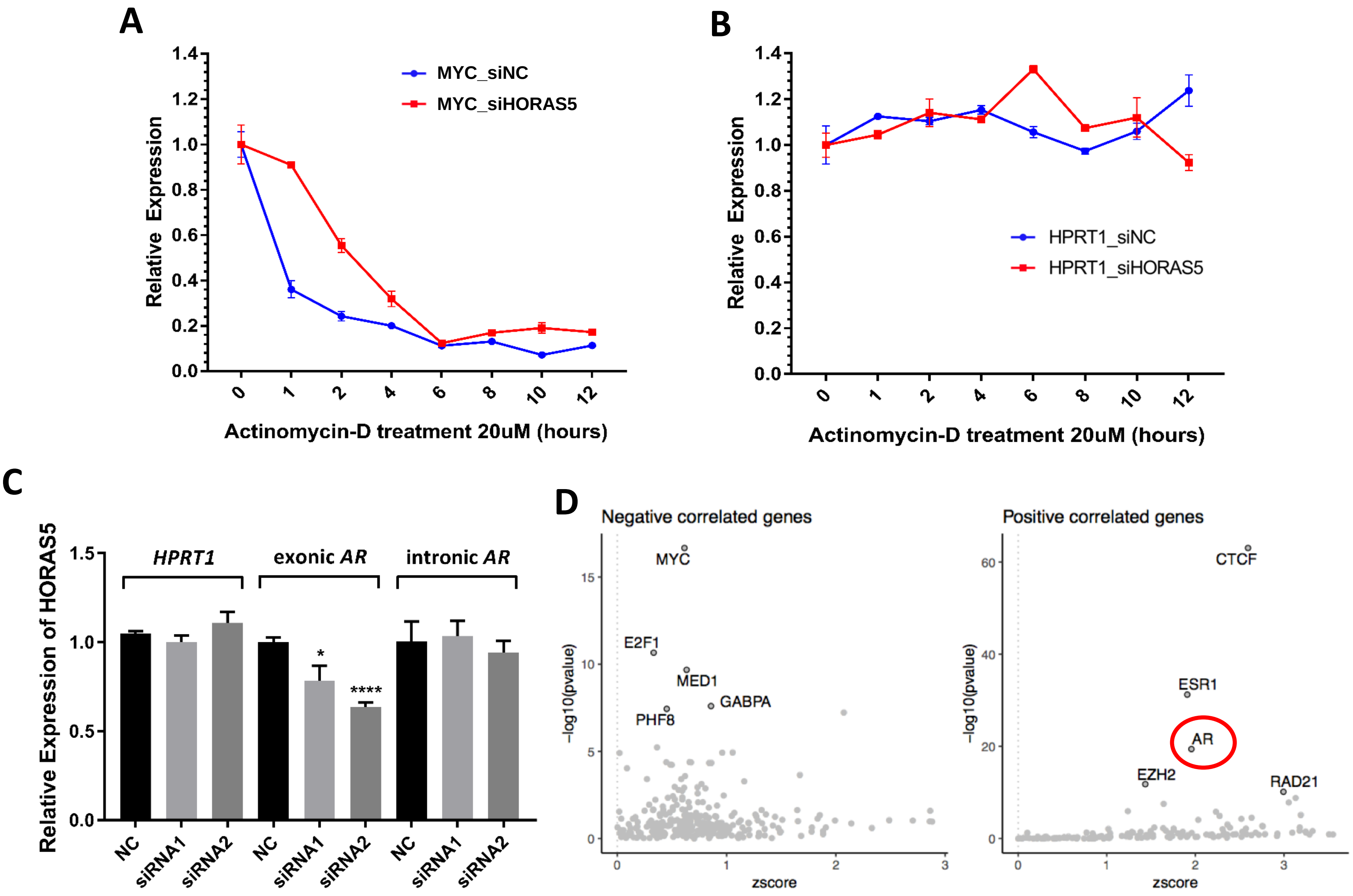

Supplementary Figure 11 | Functional regulation of AR activity by *HORAS5*. (A,B) Relative expression (qPCR) of **(A)** *MYC* or **(B)** *HPRT1* mRNA in LNCaP treated with either *HORAS5*-targeting or control siRNA (5nM for 24h), followed by treatment with the transcriptional inhibitor Actinomycin-D (20uM) for increasing durations. **(C)** Relative expression (qPCR) of mature (exonic) or premature (intronic) AR RNA in LNCaP cells treated with either control or HORAS5-targeting siRNA (5nM) for 72h. *$p<0.05$; ****$p<0.0001$ (two-way ANOVA and Tukey's test). **(D)** BART transcription factor prediction results of genes either negatively or positively correlated with *HORAS5* expression in the TCGA PCa tumors (n=499; data accessed through cBioportal). AR is highlighted in red.
