# Supplementary material for: The long noncoding RNA HORAS5 mediates castration‐resistant prostate cancer survival by activating the androgen receptor transcriptional program
Source: Mol Oncol. 2019 Mar 5;13(5):1121–36. doi: 10.1002/1878-0261.12471 (PMC6487714; doi:10.1002/1878-0261.12471)
Supplement: Supplementary file 12 — Fig. S12. HORAS5‐miRNA interaction predictions. [file MOL2-13-1121-s012.pdf]

A

ENSG00000226935.2 (*HORAS5*) (lncRNA, intergenic)							
microRNA family	Seed position	Seed type	Transcript region	Repeat	Conservation		
					Primates	Mammals	Other vert.
miR-9/9ab	chr21:29911987	8-mer	ncRNA	yes	78%	0%	0%
miR-139-5p	chr21:29912392	8-mer	ncRNA	no	0%	0%	0%
miR-145	chr21:29912166	7-mer-A1	ncRNA	yes	22%	0%	0%
miR-18ab/4735-3p	chr21:29912073	7-mer-m8	ncRNA	yes	33%	0%	0%
miR-194	chr21:29912154	7-mer-A1	ncRNA	yes	56%	0%	0%
miR-199ab-5p	chr21:29912165	7-mer-A1	ncRNA	yes	22%	0%	0%
miR-205/205ab	chr21:29911944	7-mer-m8	ncRNA	yes	56%	0%	0%
miR-208ab/208ab-3p	chr21:29912579	7-mer-A1	ncRNA	yes	33%	0%	0%
miR-214/761/3619-5p	chr21:29912066	7-mer-m8	ncRNA	yes	67%	0%	0%
miR-23abc/23b-3p	chr21:29911873	7-mer-m8	ncRNA	yes	56%	0%	0%
miR-23abc/23b-3p	chr21:29911935	7-mer-A1	ncRNA	yes	56%	0%	0%
miR-26ab/1297/4465	chr21:29911846	7-mer-m8	ncRNA	yes	44%	0%	0%
miR-27abc/27a-3p	chr21:29911886	7-mer-A1	ncRNA	yes	33%	0%	0%
miR-27abc/27a-3p	chr21:29912104	7-mer-m8	ncRNA	yes	78%	0%	0%
miR-30abcdef/30abe-5p/384-5p	chr21:29911665	7-mer-A1	ncRNA	no	44%	0%	0%
miR-33a-3p/365/365-3p	chr21:29912518	7-mer-m8	ncRNA	no	33%	0%	0%
miR-128/128ab	chr21:29912103	7-mer-A1	ncRNA	yes	78%	0%	0%
miR-499-5p	chr21:29912579	8-mer	ncRNA	yes	33%	0%	0%

B

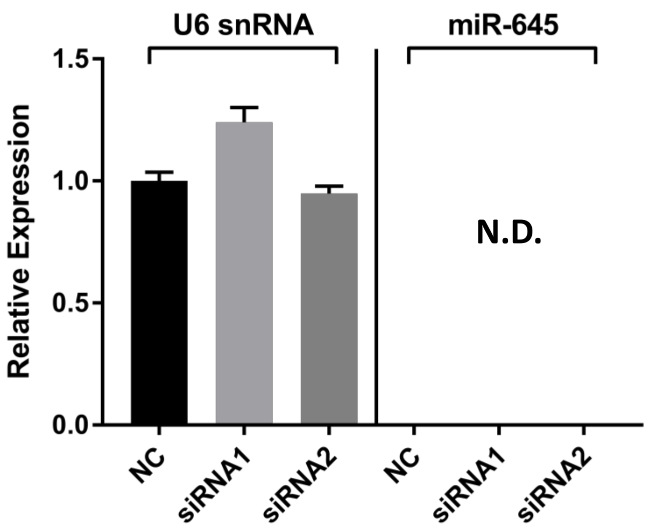

Supplementary Figure 12 | *HORAS5*-miRNA interaction predictions. (A) Table adapted from miRcode.org representing potential transcriptome-wide *HORAS5*-miRNA interactions based on UCSC GRCh37/hg19 genome assembly. **(B)** Relative expression (qPCR) of *U6 snRNA* and miR-645 in LNCaP cells treated with either *HORAS5*-targeting or control siRNAs for 72hrs. Data shown as a representative replicate.
