# Supplementary material for: The long noncoding RNA HORAS5 mediates castration‐resistant prostate cancer survival by activating the androgen receptor transcriptional program
Source: Mol Oncol. 2019 Mar 5;13(5):1121–36. doi: 10.1002/1878-0261.12471 (PMC6487714; doi:10.1002/1878-0261.12471)
Supplement: Supplementary file 13 — Table S1. LincRNAs up‐regulated in LTL‐313BR (hormone‐independent) vs LTL‐313B (hormone‐dependent). [file MOL2-13-1121-s013.pdf]

Supplemental Table 1: LincRNAs up-regulated in LTL-313BR (hormone-independent) vs LTL-313B (hormone-dependent)

GENE ID	Gene Symbol	Gene Type	LTL-313B (RPKM)	LTL-313BR (RPKM)	fold-change (BR/B)	log2fold-change (BR/B)	HORAS ID
ENSG00000255193.1	RP11-945A11.1	lincRNA	0.9895	62.6646	63.33	5.98	*HORAS1*
ENSG00000232855.2	AF131217.1	lincRNA	0.5515	20.3796	36.95	5.21	*HORAS2*
ENSG00000236532.1	AL035610.2	lincRNA	1.5896	52.1518	32.81	5.04	*HORAS3*
ENSG00000178457.3	LINC00314	lincRNA	0.4380	5.5459	12.66	3.66	*HORAS4*
ENSG00000226935.2	LINC00161	lincRNA	0.7137	8.9314	12.51	3.65	*HORAS5*
ENSG00000225298.1	LINC00113	lincRNA	0.5839	5.6462	9.67	3.27	*HORAS6*
ENSG00000272216.1	LL22NC03-2H8.5	lincRNA	3.6172	30.8479	8.53	3.09	*HORAS7*
ENSG00000263427.1	RP11-599B13.3	lincRNA	1.5085	12.6955	8.42	3.07	*HORAS8*
ENSG00000234083.1	AJ006995.3	lincRNA	0.4542	3.7196	8.19	3.03	*HORAS9*
ENSG00000260896.1	RP11-314O13.1	lincRNA	1.5896	12.4951	7.86	2.97	*HORAS10*
ENSG00000227683.1	RP11-445N18.5	lincRNA	1.4599	11.1587	7.64	2.93	*HORAS11*
ENSG00000272168.1	CASC15	lincRNA	0.6002	4.2430	7.07	2.82	*HORAS12*
ENSG00000250266.1	RP11-789C1.1	lincRNA	0.6813	4.1873	6.15	2.62	*HORAS13*
ENSG00000244649.2	CTD-2377D24.6	lincRNA	0.7786	4.6383	5.96	2.57	*HORAS14*
ENSG00000255418.1	RP11-266A24.1	lincRNA	0.8759	4.9501	5.65	2.50	*HORAS15*
ENSG00000225783.2	MIAT	lincRNA	1.8330	10.3569	5.65	2.50	*HORAS16*
ENSG00000239268.2	RP11-384F7.2	lincRNA	1.9303	10.2900	5.33	2.41	*HORAS17*
ENSG00000260884.1	AC009120.5	lincRNA	0.5191	2.7618	5.32	2.41	*HORAS18*
ENSG00000234052.1	AP001607.1	lincRNA	0.4055	2.0936	5.16	2.37	*HORAS19*
ENSG00000231236.2	AP001604.3	lincRNA	0.4542	2.3052	5.08	2.34	*HORAS20*
ENSG00000258188.1	RP11-146E13.4	lincRNA	0.9084	4.5993	5.06	2.34	*HORAS21*
ENSG00000261104.1	RP11-734K21.5	lincRNA	0.6164	3.0959	5.02	2.33	*HORAS22*
ENSG00000254861.1	RP11-945A11.2	lincRNA	0.4055	2.0268	5.00	2.32	*HORAS23*
ENSG00000237567.1	RP3-359N14.2	lincRNA	0.4380	2.1772	4.97	2.31	*HORAS24*
ENSG00000268362.1	CTD-2017D11.1	lincRNA	2.6115	12.7456	4.88	2.29	*HORAS25*
ENSG00000215386.6	LINC00478	lincRNA	3.1144	14.4718	4.65	2.22	*HORAS26*
ENSG00000251321.1	PCAT4	lincRNA	0.4217	1.9433	4.61	2.20	*HORAS27*
ENSG00000247095.2	MIR210HG	lincRNA	10.4462	47.1182	4.51	2.17	*HORAS28*
ENSG00000228741.2	RP11-309I15.1	lincRNA	1.3463	6.0025	4.46	2.16	*HORAS29*
ENSG00000205662.2	RP11-706O15.7	lincRNA	0.4217	1.8208	4.32	2.11	*HORAS30*
ENSG00000234630.1	LL22NC03-2H8.4	lincRNA	4.1525	17.5621	4.23	2.08	*HORAS31*
ENSG00000261069.2	SNORD116-20	lincRNA	1.1517	4.7998	4.17	2.06	*HORAS32*
ENSG00000228434.1	AC004951.6	lincRNA	0.6651	2.6950	4.05	2.02	*HORAS33*
ENSG00000215244.2	RP11-563J2.2	lincRNA	0.6164	2.4890	4.04	2.01	*HORAS34*
ENSG00000235499.1	AC073046.25	lincRNA	1.2003	4.7163	3.93	1.97	*HORAS35*
ENSG00000261770.1	CTC-459F4.1	lincRNA	7.0560	27.6072	3.91	1.97	*HORAS36*
ENSG00000237094.7	RP4-669L17.10	lincRNA	1.2003	4.6105	3.84	1.94	*HORAS37*
ENSG00000267350.2	RP1-178F10.3	lincRNA	12.3116	46.6449	3.79	1.92	*HORAS38*
ENSG00000250387.2	RP11-136K7.2	lincRNA	0.9084	3.3242	3.66	1.87	*HORAS39*
ENSG00000237807.3	RP11-400K9.4	lincRNA	1.7356	6.2809	3.62	1.86	*HORAS40*
ENSG00000262745.1	CTD-2377D24.8	lincRNA	0.6164	2.2161	3.60	1.85	*HORAS41*
ENSG00000233878.1	AC073133.1	lincRNA	4.1201	14.8003	3.59	1.84	*HORAS42*
ENSG00000255989.1	RP11-711M9.1	lincRNA	0.6164	2.2050	3.58	1.84	*HORAS43*
ENSG00000268658.1	LINC00664	lincRNA	1.2814	4.5158	3.52	1.82	*HORAS44*
ENSG00000261275.1	RP11-760D2.11	lincRNA	2.9197	10.2344	3.51	1.81	*HORAS45*
ENSG00000254084.1	KB-1930G5.4	lincRNA	0.7624	2.5892	3.40	1.76	*HORAS46*
ENSG00000197332.7	ZNF833P	lincRNA	0.8921	3.0068	3.37	1.75	*HORAS47*

ENSG00000213793.3	ZNF888	lincRNA	1.2490	4.0982	3.28	1.71	*HORAS48*
ENSG00000235373.1	RP11-206L10.3	lincRNA	0.4704	1.5313	3.26	1.70	*HORAS49*
ENSG00000257151.1	PWAR6	lincRNA	17.8915	57.8537	3.23	1.69	*HORAS50*
ENSG00000261452.1	RP11-509E16.1	lincRNA	1.0544	3.3910	3.22	1.69	*HORAS51*
ENSG00000261400.1	AC011525.2	lincRNA	1.8005	5.7353	3.19	1.67	*HORAS52*
ENSG00000258593.2	CTD-3051D23.4	lincRNA	0.7624	2.4166	3.17	1.66	*HORAS53*
ENSG00000261804.1	RP11-44F14.2	lincRNA	2.0114	6.2197	3.09	1.63	*HORAS54*
ENSG00000246465.1	RP11-57A19.2	lincRNA	1.5248	4.6884	3.07	1.62	*HORAS55*
ENSG00000235609.4	AF127936.7	lincRNA	3.5686	10.8636	3.04	1.61	*HORAS56*
ENSG00000260337.2	RP11-386M24.6	lincRNA	2.1249	6.4257	3.02	1.60	*HORAS57*
