# Supplementary material for: The long noncoding RNA HORAS5 mediates castration‐resistant prostate cancer survival by activating the androgen receptor transcriptional program
Source: Mol Oncol. 2019 Mar 5;13(5):1121–36. doi: 10.1002/1878-0261.12471 (PMC6487714; doi:10.1002/1878-0261.12471)
Supplement: Supplementary file 14 — Table S2. LincRNAs down‐regulated in LTL‐313BR (hormone‐independent) vs LTL‐313B (hormone‐dependent). [file MOL2-13-1121-s014.pdf]

**Supplemental Table 2: LincRNAs down-regulated in LTL-313BR (hormone-independent) vs LTL-313B (hormone-dependent)**

| GENE ID | Gene Symbol | Gene Type | LTL-313B (RPKM) | LTL-313BR (RPKM) | fold-change (BR/B) | log2fold-change (BR/B) | HORAS ID |
|---|---|---|---|---|---|---|---|
| ENSG00000236824.1 | BCYRN1 | lincRNA | 1378.8979 | 32.6575 | 0.02 | -5.40 | *HORAS58* |
| ENSG00000260260.1 | RP11-304L19.5 | lincRNA | 274.6182 | 11.6487 | 0.04 | -4.56 | *HORAS59* |
| ENSG00000232388.2 | LINC00493 | lincRNA | 111.8749 | 7.4057 | 0.07 | -3.92 | *HORAS60* |
| ENSG00000255198.3 | SNHG9 | lincRNA | 13.9986 | 1.0580 | 0.08 | -3.73 | *HORAS61* |
| ENSG00000254139.1 | CTD-2339F6.1 | lincRNA | 2.1249 | 0.2227 | 0.10 | -3.25 | *HORAS62* |
| ENSG00000245526.4 | LINC00461 | lincRNA | 12.1494 | 1.4533 | 0.12 | -3.06 | *HORAS63* |
| ENSG00000270038.1 | RP11-1070N10.7 | lincRNA | 1.0219 | 0.1503 | 0.15 | -2.76 | *HORAS64* |
| ENSG00000175701.6 | LINC00116 | lincRNA | 7.1209 | 1.0580 | 0.15 | -2.75 | *HORAS65* |
| ENSG00000273406.1 | RP11-114O18.1 | lincRNA | 1.3950 | 0.2172 | 0.16 | -2.68 | *HORAS66* |
| ENSG00000180279.5 | CTD-2568A17.5 | lincRNA | 3.6010 | 0.5791 | 0.16 | -2.64 | *HORAS67* |
| ENSG00000272048.1 | RP11-458N5.1 | lincRNA | 4.8176 | 0.8241 | 0.17 | -2.55 | *HORAS68* |
| ENSG00000228065.6 | RP11-222A11.1 | lincRNA | 0.9570 | 0.1670 | 0.17 | -2.52 | *HORAS69* |
| ENSG00000249599.1 | RP11-168E14.1 | lincRNA | 6.2775 | 1.1359 | 0.18 | -2.47 | *HORAS70* |
| ENSG00000261005.1 | CTB-58E17.1 | lincRNA | 4.3958 | 0.8464 | 0.19 | -2.38 | *HORAS71* |
| ENSG00000182165.13 | TP53TG1 | lincRNA | 44.8181 | 8.7978 | 0.20 | -2.35 | *HORAS72* |
| ENSG00000225760.1 | LINC00431 | lincRNA | 1.8654 | 0.3731 | 0.20 | -2.32 | *HORAS73* |
| ENSG00000272663.1 | RP11-191L17.1 | lincRNA | 0.9732 | 0.1949 | 0.20 | -2.32 | *HORAS74* |
| ENSG00000214546.3 | AC087491.2 | lincRNA | 2.3682 | 0.4956 | 0.21 | -2.26 | *HORAS75* |
| ENSG00000229227.4 | RP11-38L15.2 | lincRNA | 1.6221 | 0.3452 | 0.21 | -2.23 | *HORAS76* |
| ENSG00000229891.1 | Z83851.1 | lincRNA | 11.5168 | 2.4779 | 0.22 | -2.22 | *HORAS77* |
| ENSG00000236081.1 | AC074389.9 | lincRNA | 11.5817 | 2.4946 | 0.22 | -2.21 | *HORAS78* |
| ENSG00000272221.1 | XXbac-BPG181B23.7 | lincRNA | 4.9149 | 1.0635 | 0.22 | -2.21 | *HORAS79* |
| ENSG00000234692.1 | RP11-445L6.3 | lincRNA | 2.2871 | 0.5123 | 0.22 | -2.16 | *HORAS80* |
| ENSG00000243491.1 | RP11-521D12.5 | lincRNA | 0.9408 | 0.2116 | 0.22 | -2.15 | *HORAS81* |
| ENSG00000271869.1 | RP11-51J9.5 | lincRNA | 0.8110 | 0.1838 | 0.23 | -2.14 | *HORAS82* |
| ENSG00000175773.8 | RP11-121M22.1 | lincRNA | 1.0219 | 0.2339 | 0.23 | -2.13 | *HORAS83* |
| ENSG00000224032.2 | EPB41L4A-AS1 | lincRNA | 28.7433 | 6.6150 | 0.23 | -2.12 | *HORAS84* |
| ENSG00000231196.3 | RP11-495P10.8 | lincRNA | 0.9084 | 0.2116 | 0.23 | -2.10 | *HORAS85* |
| ENSG00000272369.1 | RP11-446N19.1 | lincRNA | 0.9084 | 0.2116 | 0.23 | -2.10 | *HORAS86* |
| ENSG00000272795.1 | RP11-602N24.3 | lincRNA | 0.7137 | 0.1670 | 0.23 | -2.10 | *HORAS87* |
| ENSG00000270673.1 | YTHDF3-AS1 | lincRNA | 5.6448 | 1.3364 | 0.24 | -2.08 | *HORAS88* |
| ENSG00000262890.1 | RP11-424M24.5 | lincRNA | 1.2166 | 0.2895 | 0.24 | -2.07 | *HORAS89* |
| ENSG00000258927.1 | RP11-1070N10.5 | lincRNA | 0.6975 | 0.1670 | 0.24 | -2.06 | *HORAS90* |
| ENSG00000152931.7 | PART1 | lincRNA | 133.1241 | 31.9726 | 0.24 | -2.06 | *HORAS91* |
| ENSG00000236206.1 | RP11-306I1.2 | lincRNA | 0.6002 | 0.1448 | 0.24 | -2.05 | *HORAS92* |
| ENSG00000226853.2 | AC010894.3 | lincRNA | 1.6383 | 0.3953 | 0.24 | -2.05 | *HORAS93* |
| ENSG00000228106.1 | RP11-452F19.3 | lincRNA | 4.6229 | 1.1248 | 0.24 | -2.04 | *HORAS94* |
| ENSG00000229233.1 | AC011891.5 | lincRNA | 1.0706 | 0.2617 | 0.24 | -2.03 | *HORAS95* |
| ENSG00000253339.1 | RP11-434I12.3 | lincRNA | 0.5677 | 0.1392 | 0.25 | -2.03 | *HORAS96* |
| ENSG00000244219.2 | GS1-259H13.2 | lincRNA | 0.9895 | 0.2450 | 0.25 | -2.01 | *HORAS97* |
| ENSG00000272711.1 | RP11-259N19.1 | lincRNA | 1.0057 | 0.2506 | 0.25 | -2.00 | *HORAS98* |
| ENSG00000259869.1 | AL022344.7 | lincRNA | 7.8184 | 1.9600 | 0.25 | -2.00 | *HORAS99* |
| ENSG00000270020.1 | RP11-463O9.9 | lincRNA | 1.0381 | 0.2617 | 0.25 | -1.99 | *HORAS100* |
| ENSG00000236129.1 | AP002856.4 | lincRNA | 0.6164 | 0.1559 | 0.25 | -1.98 | *HORAS101* |
| ENSG00000268649.2 | MIR296 | lincRNA | 0.6164 | 0.1559 | 0.25 | -1.98 | *HORAS102* |
| ENSG00000272452.1 | RP11-391M1.4 | lincRNA | 25.4504 | 6.4703 | 0.25 | -1.98 | *HORAS103* |
| ENSG00000270235.1 | RP11-122K13.14 | lincRNA | 0.5677 | 0.1448 | 0.26 | -1.97 | *HORAS104* |
| ENSG00000228959.1 | RP5-1121H13.3 | lincRNA | 0.6326 | 0.1615 | 0.26 | -1.97 | *HORAS105* |
| ENSG00000235532.1 | LINC00402 | lincRNA | 1.5248 | 0.3898 | 0.26 | -1.97 | *HORAS106* |

| | | | | | | | |
|---|---|---|---|---|---|---|---|
| ENSG00000267089.1 | CTC-499B15.8 | lincRNA | 1.0381 | 0.2673 | 0.26 | -1.96 | *HORAS107* |
| ENSG00000226770.1 | AC000124.1 | lincRNA | 3.3901 | 0.8742 | 0.26 | -1.96 | *HORAS108* |
| ENSG00000225255.2 | LA16c-83F12.6 | lincRNA | 17.4698 | 4.5102 | 0.26 | -1.95 | *HORAS109* |
| ENSG00000234182.1 | RP11-118K6.2 | lincRNA | 0.6813 | 0.1782 | 0.26 | -1.93 | *HORAS110* |
| ENSG00000270988.1 | RP11-439C15.5 | lincRNA | 0.6164 | 0.1615 | 0.26 | -1.93 | *HORAS111* |
| ENSG00000272525.1 | RP11-79P5.9 | lincRNA | 1.2003 | 0.3174 | 0.26 | -1.92 | *HORAS112* |
| ENSG00000267440.1 | CTC-501O10.1 | lincRNA | 0.5677 | 0.1503 | 0.26 | -1.92 | *HORAS113* |
| ENSG00000224961.1 | RP1-278O22.1 | lincRNA | 1.3463 | 0.3619 | 0.27 | -1.90 | *HORAS114* |
| ENSG00000223715.1 | RP11-71G7.1 | lincRNA | 0.6813 | 0.1838 | 0.27 | -1.89 | *HORAS115* |
| ENSG00000259711.1 | CTD-3032H12.2 | lincRNA | 0.6813 | 0.1838 | 0.27 | -1.89 | *HORAS116* |
| ENSG00000260931.1 | RP11-65L3.1 | lincRNA | 1.0706 | 0.2895 | 0.27 | -1.89 | *HORAS117* |
| ENSG00000246774.1 | AC004051.2 | lincRNA | 0.5515 | 0.1503 | 0.27 | -1.88 | *HORAS118* |
| ENSG00000259553.2 | MIR1302-10 | lincRNA | 0.5515 | 0.1503 | 0.27 | -1.88 | *HORAS119* |
| ENSG00000244459.2 | RP11-1398P2.1 | lincRNA | 0.7948 | 0.2172 | 0.27 | -1.87 | *HORAS120* |
| ENSG00000267067.1 | CTB-75G16.3 | lincRNA | 0.7948 | 0.2172 | 0.27 | -1.87 | *HORAS121* |
| ENSG00000228526.2 | RP3-510D11.1 | lincRNA | 0.6813 | 0.1893 | 0.28 | -1.85 | *HORAS122* |
| ENSG00000255992.1 | RP11-417L19.4 | lincRNA | 0.6813 | 0.1893 | 0.28 | -1.85 | *HORAS123* |
| ENSG00000226965.1 | AC003088.1 | lincRNA | 0.6002 | 0.1670 | 0.28 | -1.85 | *HORAS124* |
| ENSG00000225667.1 | LINC00505 | lincRNA | 0.6164 | 0.1726 | 0.28 | -1.84 | *HORAS125* |
| ENSG00000253839.1 | RP11-431D12.1 | lincRNA | 0.5353 | 0.1503 | 0.28 | -1.83 | *HORAS126* |
| ENSG00000270457.1 | RP11-467C18.1 | lincRNA | 0.6326 | 0.1782 | 0.28 | -1.83 | *HORAS127* |
| ENSG00000234675.1 | RP11-242F11.2 | lincRNA | 0.5677 | 0.1615 | 0.28 | -1.81 | *HORAS128* |
| ENSG00000273437.1 | RP11-434H6.7 | lincRNA | 1.5248 | 0.4343 | 0.28 | -1.81 | *HORAS129* |
| ENSG00000228792.2 | RP11-354K1.2 | lincRNA | 0.4866 | 0.1392 | 0.29 | -1.81 | *HORAS130* |
| ENSG00000232140.1 | AC073257.1 | lincRNA | 0.4866 | 0.1392 | 0.29 | -1.81 | *HORAS131* |
| ENSG00000248673.1 | CTC-419K13.1 | lincRNA | 0.4866 | 0.1392 | 0.29 | -1.81 | *HORAS132* |
| ENSG00000248859.2 | RP11-375B1.3 | lincRNA | 0.4866 | 0.1392 | 0.29 | -1.81 | *HORAS133* |
| ENSG00000253416.1 | RP11-48D4.2 | lincRNA | 0.4866 | 0.1392 | 0.29 | -1.81 | *HORAS134* |
| ENSG00000225353.2 | RP11-292F9.1 | lincRNA | 0.5839 | 0.1670 | 0.29 | -1.81 | *HORAS135* |
| ENSG00000258538.1 | RP11-753D20.3 | lincRNA | 0.4866 | 0.1392 | 0.29 | -1.81 | *HORAS136* |
| ENSG00000225532.1 | XX-C2158C6.3 | lincRNA | 0.7948 | 0.2283 | 0.29 | -1.80 | *HORAS137* |
| ENSG00000236743.1 | RP5-857K21.15 | lincRNA | 0.5028 | 0.1448 | 0.29 | -1.80 | *HORAS138* |
| ENSG00000254153.1 | CTA-398F10.2 | lincRNA | 0.6164 | 0.1782 | 0.29 | -1.79 | *HORAS139* |
| ENSG00000253844.1 | RP11-546K22.1 | lincRNA | 0.6164 | 0.1782 | 0.29 | -1.79 | *HORAS140* |
| ENSG00000262094.1 | AC139099.5 | lincRNA | 0.8273 | 0.2394 | 0.29 | -1.79 | *HORAS141* |
| ENSG00000265369.2 | U3 | lincRNA | 5.0447 | 1.4644 | 0.29 | -1.78 | *HORAS142* |
| ENSG00000182586.3 | LINC00334 | lincRNA | 0.6488 | 0.1893 | 0.29 | -1.78 | *HORAS143* |
| ENSG00000254605.1 | RP11-626H12.2 | lincRNA | 0.5515 | 0.1615 | 0.29 | -1.77 | *HORAS144* |
| ENSG00000271943.1 | RP11-222K16.1 | lincRNA | 0.5677 | 0.1670 | 0.29 | -1.76 | *HORAS145* |
| ENSG00000255983.1 | RP11-1038A11.1 | lincRNA | 2.0925 | 0.6181 | 0.30 | -1.76 | *HORAS146* |
| ENSG00000267321.1 | RP11-1094M14.11 | lincRNA | 7.6887 | 2.2718 | 0.30 | -1.76 | *HORAS147* |
| ENSG00000271991.1 | RP11-79O8.1 | lincRNA | 0.5839 | 0.1726 | 0.30 | -1.76 | *HORAS148* |
| ENSG00000240980.1 | RP11-734K21.4 | lincRNA | 0.4704 | 0.1392 | 0.30 | -1.76 | *HORAS149* |
| ENSG00000224568.1 | AC096669.3 | lincRNA | 0.4704 | 0.1392 | 0.30 | -1.76 | *HORAS150* |
| ENSG00000225105.2 | LINC01076 | lincRNA | 0.4704 | 0.1392 | 0.30 | -1.76 | *HORAS151* |
| ENSG00000231817.3 | RP11-189B4.6 | lincRNA | 0.4704 | 0.1392 | 0.30 | -1.76 | *HORAS152* |
| ENSG00000272515.1 | RP11-29P20.1 | lincRNA | 0.4704 | 0.1392 | 0.30 | -1.76 | *HORAS153* |
| ENSG00000227467.3 | RP11-169D4.1 | lincRNA | 0.4866 | 0.1448 | 0.30 | -1.75 | *HORAS154* |
| ENSG00000249094.2 | RP1-7G5.6 | lincRNA | 0.4866 | 0.1448 | 0.30 | -1.75 | *HORAS155* |
| ENSG00000223379.1 | RP11-374M1.4 | lincRNA | 0.5028 | 0.1503 | 0.30 | -1.74 | *HORAS156* |
| ENSG00000176840.7 | MIR7-3HG | lincRNA | 0.5028 | 0.1503 | 0.30 | -1.74 | *HORAS157* |
| ENSG00000229647.1 | AC007879.7 | lincRNA | 0.5191 | 0.1559 | 0.30 | -1.74 | *HORAS158* |
| ENSG00000238837.3 | RP11-646E18.2 | lincRNA | 0.5191 | 0.1559 | 0.30 | -1.74 | *HORAS159* |
| ENSG00000272853.1 | RP11-398C13.6 | lincRNA | 3.5686 | 1.0747 | 0.30 | -1.73 | *HORAS160* |
| ENSG00000251443.1 | RP11-113I22.1 | lincRNA | 0.5353 | 0.1615 | 0.30 | -1.73 | *HORAS161* |

| | | | | | | | |
|---|---|---|---|---|---|---|---|
| ENSG00000272192.1 | CTD-2532N20.1 | lincRNA | 0.5353 | 0.1615 | 0.30 | -1.73 | *HORAS162* |
| ENSG00000267421.2 | AC005498.3 | lincRNA | 0.8110 | 0.2450 | 0.30 | -1.73 | *HORAS163* |
| ENSG00000253821.1 | RP11-246K15.1 | lincRNA | 27.4780 | 8.3022 | 0.30 | -1.73 | *HORAS164* |
| ENSG00000242147.1 | RP13-463N16.6 | lincRNA | 0.5515 | 0.1670 | 0.30 | -1.72 | *HORAS165* |
| ENSG00000227718.1 | AC016730.1 | lincRNA | 6.0990 | 1.8486 | 0.30 | -1.72 | *HORAS166* |
| ENSG00000269942.1 | RP11-29B2.5 | lincRNA | 0.8435 | 0.2561 | 0.30 | -1.72 | *HORAS167* |
| ENSG00000272275.1 | RP11-791G15.2 | lincRNA | 6.0504 | 1.8375 | 0.30 | -1.72 | *HORAS168* |
| ENSG00000271926.1 | CTD-2376I4.1 | lincRNA | 0.5677 | 0.1726 | 0.30 | -1.72 | *HORAS169* |
| ENSG00000272338.1 | RP11-722E23.2 | lincRNA | 0.7137 | 0.2172 | 0.30 | -1.72 | *HORAS170* |
| ENSG00000228022.1 | HCG20 | lincRNA | 0.5839 | 0.1782 | 0.31 | -1.71 | *HORAS171* |
| ENSG00000231535.1 | LINC00278 | lincRNA | 0.7299 | 0.2227 | 0.31 | -1.71 | *HORAS172* |
| ENSG00000260360.1 | RP11-533E19.5 | lincRNA | 0.6002 | 0.1838 | 0.31 | -1.71 | *HORAS173* |
| ENSG00000248161.1 | RP11-499E18.1 | lincRNA | 0.6002 | 0.1838 | 0.31 | -1.71 | *HORAS174* |
| ENSG00000228397.1 | RP1-224A6.3 | lincRNA | 0.4542 | 0.1392 | 0.31 | -1.71 | *HORAS175* |
| ENSG00000237292.1 | RP11-540K16.1 | lincRNA | 0.4542 | 0.1392 | 0.31 | -1.71 | *HORAS176* |
| ENSG00000236452.1 | AC123023.1 | lincRNA | 0.4542 | 0.1392 | 0.31 | -1.71 | *HORAS177* |
| ENSG00000225647.1 | AC005487.2 | lincRNA | 1.3625 | 0.4176 | 0.31 | -1.71 | *HORAS178* |
| ENSG00000253154.1 | CTA-392E5.1 | lincRNA | 0.4542 | 0.1392 | 0.31 | -1.71 | *HORAS179* |
| ENSG00000256084.1 | RP11-134N1.2 | lincRNA | 0.4542 | 0.1392 | 0.31 | -1.71 | *HORAS180* |
| ENSG00000257431.1 | RP11-263K4.3 | lincRNA | 0.4542 | 0.1392 | 0.31 | -1.71 | *HORAS181* |
| ENSG00000258942.1 | RP11-255G12.2 | lincRNA | 0.4542 | 0.1392 | 0.31 | -1.71 | *HORAS182* |
| ENSG00000258383.1 | CTD-2200A16.1 | lincRNA | 0.4542 | 0.1392 | 0.31 | -1.71 | *HORAS183* |
| ENSG00000260834.1 | RP11-256I9.2 | lincRNA | 0.4542 | 0.1392 | 0.31 | -1.71 | *HORAS184* |
| ENSG00000264174.1 | RP11-212E8.1 | lincRNA | 0.4542 | 0.1392 | 0.31 | -1.71 | *HORAS185* |
| ENSG00000272715.1 | RP4-753F5.1 | lincRNA | 0.4704 | 0.1448 | 0.31 | -1.70 | *HORAS186* |
| ENSG00000250098.1 | RP11-22A3.1 | lincRNA | 0.4704 | 0.1448 | 0.31 | -1.70 | *HORAS187* |
| ENSG00000250697.1 | CTD-2066L21.3 | lincRNA | 0.4704 | 0.1448 | 0.31 | -1.70 | *HORAS188* |
| ENSG00000271897.1 | RP11-679B17.2 | lincRNA | 0.4704 | 0.1448 | 0.31 | -1.70 | *HORAS189* |
| ENSG00000255314.1 | RP11-702F3.3 | lincRNA | 0.4704 | 0.1448 | 0.31 | -1.70 | *HORAS190* |
| ENSG00000258066.1 | RP11-781A6.1 | lincRNA | 0.4704 | 0.1448 | 0.31 | -1.70 | *HORAS191* |
| ENSG00000257958.1 | RP11-25E2.1 | lincRNA | 0.4704 | 0.1448 | 0.31 | -1.70 | *HORAS192* |
| ENSG00000234503.1 | KB-1592A4.14 | lincRNA | 0.4704 | 0.1448 | 0.31 | -1.70 | *HORAS193* |
| ENSG00000228127.1 | RP11-12L8.1 | lincRNA | 0.4866 | 0.1503 | 0.31 | -1.69 | *HORAS194* |
| ENSG00000226302.1 | RP11-528N21.1 | lincRNA | 0.4866 | 0.1503 | 0.31 | -1.69 | *HORAS195* |
| ENSG00000261462.1 | CTA-254O6.1 | lincRNA | 0.4866 | 0.1503 | 0.31 | -1.69 | *HORAS196* |
| ENSG00000258279.2 | LINC00592 | lincRNA | 0.4866 | 0.1503 | 0.31 | -1.69 | *HORAS197* |
| ENSG00000237772.1 | AC092620.3 | lincRNA | 0.6651 | 0.2060 | 0.31 | -1.69 | *HORAS198* |
| ENSG00000225982.1 | RP11-538D16.3 | lincRNA | 0.5028 | 0.1559 | 0.31 | -1.69 | *HORAS199* |
| ENSG00000270697.1 | RP11-381K20.4 | lincRNA | 0.5028 | 0.1559 | 0.31 | -1.69 | *HORAS200* |
| ENSG00000245812.2 | RP11-175K6.1 | lincRNA | 0.5028 | 0.1559 | 0.31 | -1.69 | *HORAS201* |
| ENSG00000273019.1 | RP11-508N22.13 | lincRNA | 0.5028 | 0.1559 | 0.31 | -1.69 | *HORAS202* |
| ENSG00000259783.1 | RP11-1006G14.2 | lincRNA | 0.5028 | 0.1559 | 0.31 | -1.69 | *HORAS203* |
| ENSG00000261447.1 | RP11-109D9.4 | lincRNA | 0.6813 | 0.2116 | 0.31 | -1.69 | *HORAS204* |
| ENSG00000272922.1 | RP11-329B9.5 | lincRNA | 0.5191 | 0.1615 | 0.31 | -1.68 | *HORAS205* |
| ENSG00000248489.1 | CTD-2007H13.3 | lincRNA | 1.0381 | 0.3230 | 0.31 | -1.68 | *HORAS206* |
| ENSG00000254288.1 | RP11-6I2.3 | lincRNA | 0.5191 | 0.1615 | 0.31 | -1.68 | *HORAS207* |
| ENSG00000261671.1 | RP11-573G6.6 | lincRNA | 0.5191 | 0.1615 | 0.31 | -1.68 | *HORAS208* |
| ENSG00000256199.1 | RP11-439H13.2 | lincRNA | 0.5191 | 0.1615 | 0.31 | -1.68 | *HORAS209* |
| ENSG00000259362.2 | RP11-307C19.1 | lincRNA | 0.5191 | 0.1615 | 0.31 | -1.68 | *HORAS210* |
| ENSG00000267224.1 | AC005498.4 | lincRNA | 0.5191 | 0.1615 | 0.31 | -1.68 | *HORAS211* |
| ENSG00000266176.1 | RP11-855A2.5 | lincRNA | 0.5353 | 0.1670 | 0.31 | -1.68 | *HORAS212* |
| ENSG00000269246.1 | CTC-246B18.10 | lincRNA | 0.5677 | 0.1782 | 0.31 | -1.67 | *HORAS213* |
| ENSG00000270761.1 | RP11-385F7.1 | lincRNA | 1.3301 | 0.4176 | 0.31 | -1.67 | *HORAS214* |
| ENSG00000224687.1 | RASAL2-AS1 | lincRNA | 0.7624 | 0.2394 | 0.31 | -1.67 | *HORAS215* |
| ENSG00000260025.1 | RP11-490M8.1 | lincRNA | 0.7624 | 0.2394 | 0.31 | -1.67 | *HORAS216* |

| | | | | | | | |
|---|---|---|---|---|---|---|---|
| ENSG00000272931.1 | RP5-943J3.2 | lincRNA | 0.5839 | 0.1838 | 0.31 | -1.67 | *HORAS217* |
| ENSG00000240219.1 | RP11-430C7.5 | lincRNA | 0.5839 | 0.1838 | 0.31 | -1.67 | *HORAS218* |
| ENSG00000231606.1 | RP11-344F13.1 | lincRNA | 0.6002 | 0.1893 | 0.32 | -1.66 | *HORAS219* |
| ENSG00000231734.4 | RP6-206I17.2 | lincRNA | 1.2003 | 0.3786 | 0.32 | -1.66 | *HORAS220* |
| ENSG00000215866.3 | RP11-426L16.8 | lincRNA | 2.4169 | 0.7628 | 0.32 | -1.66 | *HORAS221* |
| ENSG00000230978.1 | LINC00160 | lincRNA | 0.6164 | 0.1949 | 0.32 | -1.66 | *HORAS222* |
| ENSG00000257698.1 | RP11-620J15.3 | lincRNA | 7.2669 | 2.3052 | 0.32 | -1.66 | *HORAS223* |
| ENSG00000272672.1 | RP11-302M6.5 | lincRNA | 0.4380 | 0.1392 | 0.32 | -1.65 | *HORAS224* |
| ENSG00000232347.1 | RP11-488L18.8 | lincRNA | 0.4380 | 0.1392 | 0.32 | -1.65 | *HORAS225* |
| ENSG00000223373.1 | AC108066.1 | lincRNA | 0.4380 | 0.1392 | 0.32 | -1.65 | *HORAS226* |
| ENSG00000251567.1 | RP11-775H9.2 | lincRNA | 0.4380 | 0.1392 | 0.32 | -1.65 | *HORAS227* |
| ENSG00000249752.1 | RP11-563M4.1 | lincRNA | 0.4380 | 0.1392 | 0.32 | -1.65 | *HORAS228* |
| ENSG00000249781.2 | CTD-2143L24.1 | lincRNA | 0.4380 | 0.1392 | 0.32 | -1.65 | *HORAS229* |
| ENSG00000249199.1 | CTD-2139B15.5 | lincRNA | 0.4380 | 0.1392 | 0.32 | -1.65 | *HORAS230* |
| ENSG00000251518.1 | CTD-2130F23.2 | lincRNA | 0.4380 | 0.1392 | 0.32 | -1.65 | *HORAS231* |
| ENSG00000248942.1 | CTD-2275D24.4 | lincRNA | 0.4380 | 0.1392 | 0.32 | -1.65 | *HORAS232* |
| ENSG00000249073.1 | CTD-2131I18.1 | lincRNA | 0.4380 | 0.1392 | 0.32 | -1.65 | *HORAS233* |
| ENSG00000226497.1 | RP11-406O16.1 | lincRNA | 0.4380 | 0.1392 | 0.32 | -1.65 | *HORAS234* |
| ENSG00000231533.1 | RP1-232L24.3 | lincRNA | 0.4380 | 0.1392 | 0.32 | -1.65 | *HORAS235* |
| ENSG00000272137.1 | RP11-177G23.2 | lincRNA | 0.4380 | 0.1392 | 0.32 | -1.65 | *HORAS236* |
| ENSG00000272905.1 | RP11-265E18.1 | lincRNA | 0.4380 | 0.1392 | 0.32 | -1.65 | *HORAS237* |
| ENSG00000254321.1 | RP11-495O10.1 | lincRNA | 0.4380 | 0.1392 | 0.32 | -1.65 | *HORAS238* |
| ENSG00000261710.1 | RP11-953B20.1 | lincRNA | 0.4380 | 0.1392 | 0.32 | -1.65 | *HORAS239* |
| ENSG00000228467.2 | RP11-402N8.1 | lincRNA | 0.4380 | 0.1392 | 0.32 | -1.65 | *HORAS240* |
| ENSG00000254434.1 | CTD-2555I5.1 | lincRNA | 0.4380 | 0.1392 | 0.32 | -1.65 | *HORAS241* |
| ENSG00000261703.1 | RP11-327F22.5 | lincRNA | 0.4380 | 0.1392 | 0.32 | -1.65 | *HORAS242* |
| ENSG00000273335.1 | RP11-61L19.2 | lincRNA | 0.4380 | 0.1392 | 0.32 | -1.65 | *HORAS243* |
| ENSG00000271784.1 | RP1-28H20.3 | lincRNA | 0.4380 | 0.1392 | 0.32 | -1.65 | *HORAS244* |
| ENSG00000236871.2 | LINC00106 | lincRNA | 0.4380 | 0.1392 | 0.32 | -1.65 | *HORAS245* |
| ENSG00000236120.2 | RP11-733O18.1 | lincRNA | 0.4380 | 0.1392 | 0.32 | -1.65 | *HORAS246* |
| ENSG00000205837.3 | LINC00487 | lincRNA | 0.4542 | 0.1448 | 0.32 | -1.65 | *HORAS247* |
| ENSG00000272180.1 | RP11-481J13.1 | lincRNA | 0.4542 | 0.1448 | 0.32 | -1.65 | *HORAS248* |
| ENSG00000224844.1 | AC107079.1 | lincRNA | 0.4542 | 0.1448 | 0.32 | -1.65 | *HORAS249* |
| ENSG00000249588.1 | CTC-537E7.1 | lincRNA | 0.4542 | 0.1448 | 0.32 | -1.65 | *HORAS250* |
| ENSG00000254038.1 | RP11-419C23.1 | lincRNA | 0.4542 | 0.1448 | 0.32 | -1.65 | *HORAS251* |
| ENSG00000253115.1 | RP11-6I2.4 | lincRNA | 0.4542 | 0.1448 | 0.32 | -1.65 | *HORAS252* |
| ENSG00000273415.1 | RP11-702B10.2 | lincRNA | 0.4542 | 0.1448 | 0.32 | -1.65 | *HORAS253* |
| ENSG00000255727.1 | RP11-508P1.2 | lincRNA | 0.4542 | 0.1448 | 0.32 | -1.65 | *HORAS254* |
| ENSG00000258977.1 | RP11-799P8.1 | lincRNA | 0.4542 | 0.1448 | 0.32 | -1.65 | *HORAS255* |
| ENSG00000267364.1 | RP11-47L3.1 | lincRNA | 0.4542 | 0.1448 | 0.32 | -1.65 | *HORAS256* |
| ENSG00000272709.1 | RP11-458D21.6 | lincRNA | 1.1517 | 0.3675 | 0.32 | -1.65 | *HORAS257* |
| ENSG00000233975.1 | RP11-288L9.1 | lincRNA | 0.4704 | 0.1503 | 0.32 | -1.65 | *HORAS258* |
| ENSG00000259928.1 | RP11-218M11.1 | lincRNA | 0.4704 | 0.1503 | 0.32 | -1.65 | *HORAS259* |
| ENSG00000269729.1 | AC006262.4 | lincRNA | 0.4704 | 0.1503 | 0.32 | -1.65 | *HORAS260* |
| ENSG00000223804.1 | RP6-206I17.1 | lincRNA | 10.2029 | 3.2630 | 0.32 | -1.64 | *HORAS261* |
| ENSG00000226281.2 | RP1-80N2.2 | lincRNA | 0.4866 | 0.1559 | 0.32 | -1.64 | *HORAS262* |
| ENSG00000229720.1 | RP3-495K2.2 | lincRNA | 0.7299 | 0.2339 | 0.32 | -1.64 | *HORAS263* |
| ENSG00000267785.1 | CTD-3194G12.2 | lincRNA | 0.4866 | 0.1559 | 0.32 | -1.64 | *HORAS264* |
| ENSG00000251665.1 | RP11-700H6.2 | lincRNA | 0.4866 | 0.1559 | 0.32 | -1.64 | *HORAS265* |
| ENSG00000249426.1 | CTC-448D22.1 | lincRNA | 0.5028 | 0.1615 | 0.32 | -1.64 | *HORAS266* |
| ENSG00000273297.1 | RP11-38M8.1 | lincRNA | 0.5028 | 0.1615 | 0.32 | -1.64 | *HORAS267* |
| ENSG00000272282.1 | RP11-222K16.2 | lincRNA | 0.5191 | 0.1670 | 0.32 | -1.64 | *HORAS268* |
| ENSG00000249396.1 | RP11-1C1.4 | lincRNA | 0.5353 | 0.1726 | 0.32 | -1.63 | *HORAS269* |
| ENSG00000234902.2 | AC007879.2 | lincRNA | 0.5515 | 0.1782 | 0.32 | -1.63 | *HORAS270* |
| ENSG00000251291.1 | RP11-400D2.3 | lincRNA | 0.5515 | 0.1782 | 0.32 | -1.63 | *HORAS271* |

| | | | | | | | |
|---|---|---|---|---|---|---|---|
| ENSG00000258100.1 | RP11-121E16.1 | lincRNA | 0.5515 | 0.1782 | 0.32 | -1.63 | *HORAS272* |
| ENSG00000225156.2 | AC012354.6 | lincRNA | 0.5677 | 0.1838 | 0.32 | -1.63 | *HORAS273* |
| ENSG00000238062.1 | AC105344.2 | lincRNA | 0.5677 | 0.1838 | 0.32 | -1.63 | *HORAS274* |
| ENSG00000185044.10 | RP11-435B5.4 | lincRNA | 6.8290 | 2.2106 | 0.32 | -1.63 | *HORAS275* |
| ENSG00000260588.1 | RP11-930P14.2 | lincRNA | 1.8492 | 0.6014 | 0.33 | -1.62 | *HORAS276* |
| ENSG00000272829.1 | XXbac-B135H6.18 | lincRNA | 1.2328 | 0.4009 | 0.33 | -1.62 | *HORAS277* |
| ENSG00000223891.1 | OSER1-AS1 | lincRNA | 5.5962 | 1.8208 | 0.33 | -1.62 | *HORAS278* |
| ENSG00000233730.1 | RP4-666F24.3 | lincRNA | 0.9570 | 0.3118 | 0.33 | -1.62 | *HORAS279* |
| ENSG00000272065.1 | U91328.20 | lincRNA | 0.6488 | 0.2116 | 0.33 | -1.62 | *HORAS280* |
| ENSG00000273403.1 | RP11-329B9.3 | lincRNA | 0.6813 | 0.2227 | 0.33 | -1.61 | *HORAS281* |
| ENSG00000257433.1 | RP1-197B17.3 | lincRNA | 1.4112 | 0.4622 | 0.33 | -1.61 | *HORAS282* |
| ENSG00000267890.1 | CTD-2126E3.4 | lincRNA | 0.7299 | 0.2394 | 0.33 | -1.61 | *HORAS283* |
| ENSG00000269961.1 | CTD-2033C11.1 | lincRNA | 0.8110 | 0.2673 | 0.33 | -1.60 | *HORAS284* |
| ENSG00000215859.4 | RP6-74O6.2 | lincRNA | 0.4217 | 0.1392 | 0.33 | -1.60 | *HORAS285* |
| ENSG00000243636.1 | RP11-164O23.7 | lincRNA | 0.4217 | 0.1392 | 0.33 | -1.60 | *HORAS286* |
| ENSG00000232679.1 | RP11-400N13.3 | lincRNA | 0.4217 | 0.1392 | 0.33 | -1.60 | *HORAS287* |
| ENSG00000234362.1 | AC104654.2 | lincRNA | 0.4217 | 0.1392 | 0.33 | -1.60 | *HORAS288* |
| ENSG00000230923.1 | LINC00309 | lincRNA | 0.4217 | 0.1392 | 0.33 | -1.60 | *HORAS289* |
| ENSG00000236780.1 | AC078941.1 | lincRNA | 0.4217 | 0.1392 | 0.33 | -1.60 | *HORAS290* |
| ENSG00000235519.1 | AC012075.2 | lincRNA | 0.4217 | 0.1392 | 0.33 | -1.60 | *HORAS291* |
| ENSG00000272774.1 | RP11-433A10.3 | lincRNA | 0.4217 | 0.1392 | 0.33 | -1.60 | *HORAS292* |
| ENSG00000242339.1 | RP11-735B13.2 | lincRNA | 0.4217 | 0.1392 | 0.33 | -1.60 | *HORAS293* |
| ENSG00000250125.1 | RP11-707A18.1 | lincRNA | 0.4217 | 0.1392 | 0.33 | -1.60 | *HORAS294* |
| ENSG00000249275.1 | RP11-364P22.2 | lincRNA | 0.4217 | 0.1392 | 0.33 | -1.60 | *HORAS295* |
| ENSG00000271334.1 | CTD-2078B5.2 | lincRNA | 0.4217 | 0.1392 | 0.33 | -1.60 | *HORAS296* |
| ENSG00000260192.1 | RP11-756H20.1 | lincRNA | 0.4217 | 0.1392 | 0.33 | -1.60 | *HORAS297* |
| ENSG00000254298.1 | CTB-17P3.4 | lincRNA | 0.4217 | 0.1392 | 0.33 | -1.60 | *HORAS298* |
| ENSG00000237530.1 | RP3-449H6.1 | lincRNA | 0.4217 | 0.1392 | 0.33 | -1.60 | *HORAS299* |
| ENSG00000272915.1 | RP11-62J1.4 | lincRNA | 0.4217 | 0.1392 | 0.33 | -1.60 | *HORAS300* |
| ENSG00000254026.1 | RP11-369E15.2 | lincRNA | 0.4217 | 0.1392 | 0.33 | -1.60 | *HORAS301* |
| ENSG00000253679.1 | KB-1410C5.2 | lincRNA | 0.4217 | 0.1392 | 0.33 | -1.60 | *HORAS302* |
| ENSG00000254227.1 | RP11-622O11.4 | lincRNA | 0.4217 | 0.1392 | 0.33 | -1.60 | *HORAS303* |
| ENSG00000227917.1 | RP11-143M1.3 | lincRNA | 0.4217 | 0.1392 | 0.33 | -1.60 | *HORAS304* |
| ENSG00000232035.1 | RP11-87N24.3 | lincRNA | 0.4217 | 0.1392 | 0.33 | -1.60 | *HORAS305* |
| ENSG00000227809.1 | RP11-171A24.2 | lincRNA | 0.4217 | 0.1392 | 0.33 | -1.60 | *HORAS306* |
| ENSG00000230109.1 | RP11-275N1.1 | lincRNA | 0.4217 | 0.1392 | 0.33 | -1.60 | *HORAS307* |
| ENSG00000235356.1 | RP11-428G2.1 | lincRNA | 0.4217 | 0.1392 | 0.33 | -1.60 | *HORAS308* |
| ENSG00000241317.1 | RP11-342M3.1 | lincRNA | 0.4217 | 0.1392 | 0.33 | -1.60 | *HORAS309* |
| ENSG00000255243.1 | CTD-2507G9.1 | lincRNA | 0.4217 | 0.1392 | 0.33 | -1.60 | *HORAS310* |
| ENSG00000254669.1 | RP5-945I17.2 | lincRNA | 0.4217 | 0.1392 | 0.33 | -1.60 | *HORAS311* |
| ENSG00000254885.1 | RP11-802F5.1 | lincRNA | 0.4217 | 0.1392 | 0.33 | -1.60 | *HORAS312* |
| ENSG00000254874.1 | RP11-676F20.1 | lincRNA | 0.4217 | 0.1392 | 0.33 | -1.60 | *HORAS313* |
| ENSG00000256427.1 | RP11-118B22.4 | lincRNA | 0.4217 | 0.1392 | 0.33 | -1.60 | *HORAS314* |
| ENSG00000256373.1 | RP11-711K1.8 | lincRNA | 0.4217 | 0.1392 | 0.33 | -1.60 | *HORAS315* |
| ENSG00000257435.1 | RP11-267D19.1 | lincRNA | 0.4217 | 0.1392 | 0.33 | -1.60 | *HORAS316* |
| ENSG00000260030.1 | RP11-686F15.2 | lincRNA | 0.4217 | 0.1392 | 0.33 | -1.60 | *HORAS317* |
| ENSG00000257477.1 | RP11-753H16.4 | lincRNA | 0.4217 | 0.1392 | 0.33 | -1.60 | *HORAS318* |
| ENSG00000258084.1 | RP11-754N21.1 | lincRNA | 0.4217 | 0.1392 | 0.33 | -1.60 | *HORAS319* |
| ENSG00000258338.1 | RP11-87P13.2 | lincRNA | 0.4217 | 0.1392 | 0.33 | -1.60 | *HORAS320* |
| ENSG00000257725.1 | RP11-654D12.3 | lincRNA | 0.4217 | 0.1392 | 0.33 | -1.60 | *HORAS321* |
| ENSG00000259124.1 | RP11-187O7.3 | lincRNA | 0.4217 | 0.1392 | 0.33 | -1.60 | *HORAS322* |
| ENSG00000259527.1 | LINC00052 | lincRNA | 0.4217 | 0.1392 | 0.33 | -1.60 | *HORAS323* |
| ENSG00000260071.1 | RP11-418I22.2 | lincRNA | 0.4217 | 0.1392 | 0.33 | -1.60 | *HORAS324* |
| ENSG00000236377.1 | AC084809.3 | lincRNA | 0.4217 | 0.1392 | 0.33 | -1.60 | *HORAS325* |
| ENSG00000264488.1 | RP11-605F20.1 | lincRNA | 0.4217 | 0.1392 | 0.33 | -1.60 | *HORAS326* |

| | | | | | | | |
|---|---|---|---|---|---|---|---|
| ENSG00000263611.1 | RP11-612A1.1 | lincRNA | 0.4217 | 0.1392 | 0.33 | -1.60 | *HORAS327* |
| ENSG00000260433.1 | RP11-202D1.2 | lincRNA | 0.4217 | 0.1392 | 0.33 | -1.60 | *HORAS328* |
| ENSG00000227075.1 | AP000472.3 | lincRNA | 0.4217 | 0.1392 | 0.33 | -1.60 | *HORAS329* |
| ENSG00000229962.1 | AP000221.1 | lincRNA | 0.4217 | 0.1392 | 0.33 | -1.60 | *HORAS330* |
| ENSG00000226527.1 | AP000289.6 | lincRNA | 0.4217 | 0.1392 | 0.33 | -1.60 | *HORAS331* |
| ENSG00000224592.1 | RP5-884C9.2 | lincRNA | 0.4380 | 0.1448 | 0.33 | -1.60 | *HORAS332* |
| ENSG00000223675.1 | RP11-86H7.6 | lincRNA | 0.4380 | 0.1448 | 0.33 | -1.60 | *HORAS333* |
| ENSG00000238122.1 | RP11-483I13.2 | lincRNA | 0.4380 | 0.1448 | 0.33 | -1.60 | *HORAS334* |
| ENSG00000234283.1 | RP11-495P10.6 | lincRNA | 0.4380 | 0.1448 | 0.33 | -1.60 | *HORAS335* |
| ENSG00000228437.1 | RP11-400N13.2 | lincRNA | 0.4380 | 0.1448 | 0.33 | -1.60 | *HORAS336* |
| ENSG00000233005.1 | AC067959.1 | lincRNA | 0.4380 | 0.1448 | 0.33 | -1.60 | *HORAS337* |
| ENSG00000242790.1 | RP11-451G4.3 | lincRNA | 0.4380 | 0.1448 | 0.33 | -1.60 | *HORAS338* |
| ENSG00000251688.1 | RP11-752L20.5 | lincRNA | 0.4380 | 0.1448 | 0.33 | -1.60 | *HORAS339* |
| ENSG00000250786.1 | SNHG18 | lincRNA | 0.4380 | 0.1448 | 0.33 | -1.60 | *HORAS340* |
| ENSG00000272403.1 | RP1-93H18.7 | lincRNA | 0.4380 | 0.1448 | 0.33 | -1.60 | *HORAS341* |
| ENSG00000228495.1 | LINC01013 | lincRNA | 0.4380 | 0.1448 | 0.33 | -1.60 | *HORAS342* |
| ENSG00000253452.1 | RP11-473J6.1 | lincRNA | 0.4380 | 0.1448 | 0.33 | -1.60 | *HORAS343* |
| ENSG00000224549.1 | RP11-370B11.3 | lincRNA | 0.4380 | 0.1448 | 0.33 | -1.60 | *HORAS344* |
| ENSG00000260100.1 | RP11-220I1.5 | lincRNA | 0.4380 | 0.1448 | 0.33 | -1.60 | *HORAS345* |
| ENSG00000256733.1 | RP11-881M11.8 | lincRNA | 0.4380 | 0.1448 | 0.33 | -1.60 | *HORAS346* |
| ENSG00000249388.1 | RP11-834C11.6 | lincRNA | 0.4380 | 0.1448 | 0.33 | -1.60 | *HORAS347* |
| ENSG00000256763.1 | RP1-116K23.1 | lincRNA | 0.4380 | 0.1448 | 0.33 | -1.60 | *HORAS348* |
| ENSG00000223626.1 | LINC01044 | lincRNA | 0.4380 | 0.1448 | 0.33 | -1.60 | *HORAS349* |
| ENSG00000260756.1 | RP11-609N14.1 | lincRNA | 0.4380 | 0.1448 | 0.33 | -1.60 | *HORAS350* |
| ENSG00000260201.1 | RP11-143N13.2 | lincRNA | 0.4380 | 0.1448 | 0.33 | -1.60 | *HORAS351* |
| ENSG00000260963.1 | RP11-297L17.2 | lincRNA | 0.4380 | 0.1448 | 0.33 | -1.60 | *HORAS352* |
| ENSG00000263489.1 | CTC-264K15.6 | lincRNA | 0.4380 | 0.1448 | 0.33 | -1.60 | *HORAS353* |
| ENSG00000264699.1 | RP11-421N8.2 | lincRNA | 0.4380 | 0.1448 | 0.33 | -1.60 | *HORAS354* |
| ENSG00000225877.1 | AC004603.4 | lincRNA | 0.4380 | 0.1448 | 0.33 | -1.60 | *HORAS355* |
| ENSG00000229484.1 | RP5-888M10.2 | lincRNA | 0.4542 | 0.1503 | 0.33 | -1.60 | *HORAS356* |
| ENSG00000272371.1 | RP11-25O10.2 | lincRNA | 0.4542 | 0.1503 | 0.33 | -1.60 | *HORAS357* |
| ENSG00000272823.1 | RP11-295M18.6 | lincRNA | 0.4542 | 0.1503 | 0.33 | -1.60 | *HORAS358* |
| ENSG00000225916.1 | AC007879.4 | lincRNA | 0.4542 | 0.1503 | 0.33 | -1.60 | *HORAS359* |
| ENSG00000242258.1 | LINC00996 | lincRNA | 0.4542 | 0.1503 | 0.33 | -1.60 | *HORAS360* |
| ENSG00000254337.1 | RP11-865I6.2 | lincRNA | 0.4542 | 0.1503 | 0.33 | -1.60 | *HORAS361* |
| ENSG00000223716.2 | RP11-113O24.3 | lincRNA | 0.4542 | 0.1503 | 0.33 | -1.60 | *HORAS362* |
| ENSG00000256686.1 | RP11-443N24.2 | lincRNA | 0.4542 | 0.1503 | 0.33 | -1.60 | *HORAS363* |
| ENSG00000240006.1 | RP11-200A1.1 | lincRNA | 0.4704 | 0.1559 | 0.33 | -1.59 | *HORAS364* |
| ENSG00000249173.1 | RP11-701P16.4 | lincRNA | 0.4704 | 0.1559 | 0.33 | -1.59 | *HORAS365* |
| ENSG00000249740.1 | CTD-2127H9.1 | lincRNA | 0.4704 | 0.1559 | 0.33 | -1.59 | *HORAS366* |
| ENSG00000253652.1 | RP11-798K23.4 | lincRNA | 0.4704 | 0.1559 | 0.33 | -1.59 | *HORAS367* |
| ENSG00000232505.2 | XXbac-BPG308J9.3 | lincRNA | 0.4704 | 0.1559 | 0.33 | -1.59 | *HORAS368* |
| ENSG00000237359.1 | RP11-509J21.2 | lincRNA | 0.4704 | 0.1559 | 0.33 | -1.59 | *HORAS369* |
| ENSG00000231193.1 | RP11-462B18.2 | lincRNA | 0.4704 | 0.1559 | 0.33 | -1.59 | *HORAS370* |
| ENSG00000226792.2 | LINC00371 | lincRNA | 0.4704 | 0.1559 | 0.33 | -1.59 | *HORAS371* |
| ENSG00000259469.1 | RP11-227D13.4 | lincRNA | 0.4704 | 0.1559 | 0.33 | -1.59 | *HORAS372* |
| ENSG00000269387.1 | RP11-298J23.8 | lincRNA | 0.4866 | 0.1615 | 0.33 | -1.59 | *HORAS373* |
| ENSG00000270638.1 | RP3-466P17.1 | lincRNA | 0.4866 | 0.1615 | 0.33 | -1.59 | *HORAS374* |
| ENSG00000226676.1 | RP11-589B3.6 | lincRNA | 0.4866 | 0.1615 | 0.33 | -1.59 | *HORAS375* |
| ENSG00000229425.1 | AJ006998.2 | lincRNA | 0.4866 | 0.1615 | 0.33 | -1.59 | *HORAS376* |
| ENSG00000269893.2 | SNHG8 | lincRNA | 89.9606 | 29.8623 | 0.33 | -1.59 | *HORAS377* |
| ENSG00000176754.8 | LINC00303 | lincRNA | 0.5028 | 0.1670 | 0.33 | -1.59 | *HORAS378* |
| ENSG00000233569.1 | RP11-500B12.1 | lincRNA | 1.5085 | 0.5011 | 0.33 | -1.59 | *HORAS379* |
| ENSG00000237248.3 | LINC00987 | lincRNA | 0.5028 | 0.1670 | 0.33 | -1.59 | *HORAS380* |
| ENSG00000259113.1 | RP11-406H23.2 | lincRNA | 0.5028 | 0.1670 | 0.33 | -1.59 | *HORAS381* |

| | | | | | | | |
|---|---|---|---|---|---|---|---|
| ENSG00000269736.1 | CTD-2521M24.11 | lincRNA | 0.5028 | 0.1670 | 0.33 | -1.59 | *HORAS382* |
| ENSG00000259763.1 | RP11-327J17.1 | lincRNA | 0.5191 | 0.1726 | 0.33 | -1.59 | *HORAS383* |
| ENSG00000227477.1 | STK4-AS1 | lincRNA | 0.5191 | 0.1726 | 0.33 | -1.59 | *HORAS384* |
| ENSG00000234944.1 | RP11-124O11.1 | lincRNA | 1.0706 | 0.3564 | 0.33 | -1.59 | *HORAS385* |
| ENSG00000270069.1 | RP6-99M1.2 | lincRNA | 0.5515 | 0.1838 | 0.33 | -1.59 | *HORAS386* |
