# Supplementary material for: The long noncoding RNA HORAS5 mediates castration‐resistant prostate cancer survival by activating the androgen receptor transcriptional program
Source: Mol Oncol. 2019 Mar 5;13(5):1121–36. doi: 10.1002/1878-0261.12471 (PMC6487714; doi:10.1002/1878-0261.12471)
Supplement: Supplementary file 15 — Table S3. List of proteins that are up‐regulated (FC ≥ 3) upon HORAS5 knockdown in LNCaP cells. [file MOL2-13-1121-s015.pdf]

Supplemental Table 3: List of proteins that are up-regulated (FC>=3) upon HORAS5 knockdown in LNCaP cells

Rank#	Protein names	Gene names	Fold Change si*HORAS5* /siNC (normalized)
1	Eukaryotic translation elongation factor 1 epsilon-1	EEF1E1	10.165
2	Probable cytosolic iron-sulfur protein assembly protein CIAO1	CIAO1	8.9602
3	Phosphoserine aminotransferase	PSAT1	6.9971
4	Elongation factor Ts, mitochondrial	TSFM	6.7498
5	Nucleolar protein 6	NOL6	6.1906
6	Zinc finger protein 512	ZNF512	6.0879
7	Transmembrane protein 205	TMEM205	5.7629
8	Mitochondrial carrier homolog 2	MTCH2	5.7424
9	Dipeptidyl peptidase 3	DPP3	5.6693
10	Ubiquitin-protein ligase E3C	UBE3C	5.2132
11	40S ribosomal protein S8	RPS8	5.1103
12	N-acetyltransferase 10	NAT10	5.0264
13	60S ribosomal protein L27	RPL27	4.9793
14	60S ribosomal protein L7a	RPL7A	4.9669
15	Probable global transcription activator SNF2L1;SWI/SNF-related matrix-associated actin-dependent regulator of chromatin subfamily A member 5	SMARCA1;SMARCA5	4.9262
16	Basic leucine zipper and W2 domain-containing protein 2	BZW2	4.8769
17	Vesicle-trafficking protein SEC22b	SEC22B	4.8385
18	Serine--tRNA ligase, mitochondrial	SARS2	4.7727
19	Very-long-chain enoyl-CoA reductase	TECR	4.6856
20	Epoxide hydrolase 1	EPHX1	4.6202
21	Prohibitin-2	PHB2	4.6193
22	40S ribosomal protein S26;Putative 40S ribosomal protein S26-like 1	RPS26;RPS26P11	4.5978
23	V-type proton ATPase subunit H	ATP6V1H	4.5671
24	60S ribosomal protein L6	RPL6	4.4989
25	Retinol dehydrogenase 11	RDH11	4.4976
26	Tricarboxylate transport protein, mitochondrial	SLC25A1	4.4325
27	Mannose-6-phosphate isomerase	MPI	4.396
28	39S ribosomal protein L3, mitochondrial	MRPL3	4.3662
29	40S ribosomal protein S2	RPS2	4.3635
30	rRNA 2-O-methyltransferase fibrillarin	FBL	4.3516
31	HEAT repeat-containing protein 5B	HEATR5B	4.3458
32	Cytochrome c oxidase subunit 2	MT-CO2	4.1739
33	7-dehydrocholesterol reductase	DHCR7	4.1476
34	Sideroflexin-3	SFXN3	4.1313
35	ATP-binding cassette sub-family F member 3	ABCF3	4.1228
36	40S ribosomal protein S9	RPS9	4.1048
37	Coatomer subunit zeta-1	COPZ1	4.0837
38	Dehydrogenase/reductase SDR family member 4	DHRS4	4.0654
39	60S ribosomal protein L4	RPL4	4.0596
40	Leucine--tRNA ligase, cytoplasmic	LARS	4.0543
41	Ubiquitin-like protein ISG15	ISG15	4.0515
42	Ras-related protein Rab-14	RAB14	4.0481
43	60S ribosomal protein L3	RPL3	4.0388
44	Glucosylceramidase	GBA	4.0203

45	Fructose-bisphosphate aldolase;Fructose-bisphosphate aldolase C	ALDOC	4.0084
46	NADH dehydrogenase [ubiquinone] flavoprotein 1, mitochondrial	NDUFV1	4.0039
47	Protein RRP5 homolog	PDCD11	3.992
48	RNA-binding protein NOB1	NOB1	3.9747
49	N-alpha-acetyltransferase 15, NatA auxiliary subunit	NAA15	3.9695
50	m7GpppX diphosphatase	DCPS	3.9589
51	Alpha-N-acetylglucosaminidase;Alpha-N-acetylglucosaminidase 82 kDa form;Alpha-N-acetylglucosaminidase 77 kDa form	NAGLU	3.954
52	Cystathionine beta-synthase	CBS	3.9425
53	Ribosomal protein L15;60S ribosomal protein L15	RPL15	3.9408
54	AP-2 complex subunit beta	AP2B1	3.9404
55	Nucleolar RNA helicase 2	DDX21	3.9296
56	Pescadillo homolog	PES1	3.9277
57	60S ribosomal protein L13a	RPL13A	3.9029
58	4-trimethylaminobutyraldehyde dehydrogenase	ALDH9A1	3.8939
59	Squamous cell carcinoma antigen recognized by T-cells 3	SART3	3.876
60	Dipeptidyl peptidase 2	DPP7	3.8637
61	Protein arginine N-methyltransferase 5;Protein arginine N-methyltransferase 5, N-terminally processed	PRMT5	3.8442
62	Pyridoxal kinase	PDXK	3.8436
63	Ras-related C3 botulinum toxin substrate 1;Ras-related C3 botulinum toxin substrate 3	RAC1;RAC3	3.8412
64	Nucleolar protein 58	NOP58	3.8194
65	Nitrilase homolog 1	NIT1	3.8148
66	39S ribosomal protein L16, mitochondrial	MRPL16	3.8099
67	Cytochrome b-c1 complex subunit 2, mitochondrial	UQCRC2	3.807
68	Dolichyl-diphosphooligosaccharide--protein glycosyltransferase 48 kDa subunit	DDOST	3.8026
69	Sideroflexin-1	SFXN1	3.8019
70	60S ribosomal protein L13	RPL13	3.7923
71	Importin-4	IPO4	3.7887
72	60S ribosomal protein L23	RPL23	3.7762
73	Ribosomal L1 domain-containing protein 1	RSL1D1	3.775
74	Bifunctional glutamate/proline--tRNA ligase;Glutamate--tRNA ligase;Proline--tRNA ligase	EPRS	3.7622
75	Isoleucine--tRNA ligase, cytoplasmic	IARS	3.7573
76	Vesicle-fusing ATPase	NSF	3.7331
77	Electron transfer flavoprotein subunit alpha, mitochondrial	ETFA	3.7254
78	Coatomer subunit alpha;Xenin;Proxenin	COPA	3.7251
79	40S ribosomal protein S27-like;40S ribosomal protein S27	RPS27;RPS27L	3.723
80	Branched-chain-amino-acid aminotransferase, mitochondrial;Branched-chain-amino-acid aminotransferase	BCAT2	3.7138
81	Arginine--tRNA ligase, cytoplasmic	RARS	3.7102
82	60S ribosomal protein L21	RPL21	3.6967
83	Nicotinate phosphoribosyltransferase	NAPRT1	3.6923
84	Leukotriene A-4 hydrolase	LTA4H	3.6864
85	T-complex protein 1 subunit gamma	CCT3	3.6809
86	WD repeat-containing protein 82	WDR82	3.679
87	Actin-related protein 2/3 complex subunit 3	ARPC3	3.6623
88	Secretory carrier-associated membrane protein 1	SCAMP1	3.6571
89	Phosphoglycerate mutase 1;Probable phosphoglycerate mutase 4	PGAM1;PGAM4	3.6562
90	Serine/threonine-protein phosphatase 2A 55 kDa regulatory subunit B alpha isoform	PPP2R2A	3.6439

91	60S ribosomal protein L7	RPL7	3.6368
92	Apoptosis-inducing factor 1, mitochondrial	AIFM1	3.6326
93	Casein kinase II subunit alpha	CSNK2A1	3.6317
94	NADH dehydrogenase [ubiquinone] iron-sulfur protein 2, mitochondrial	NDUFS2	3.6145
95	60S ribosomal protein L27a	RPL27A	3.6084
96	Citrate synthase;Citrate synthase, mitochondrial	CS	3.567
97	ATP synthase subunit gamma;ATP synthase subunit gamma, mitochondrial	ATP5C1	3.561
98	DNA-dependent protein kinase catalytic subunit	PRKDC	3.5587
99	Structural maintenance of chromosomes flexible hinge domain-containing protein 1	SMCHD1	3.5576
100	40S ribosomal protein S6	RPS6	3.5457
101	6-phosphofructokinase, liver type	PFKL	3.5359
102	N-acetylgalactosaminyltransferase 7	GALNT7	3.5261
103	Guanidinoacetate N-methyltransferase	GAMT	3.5139
104	Procollagen-lysine,2-oxoglutarate 5-dioxygenase 1	PLOD1	3.5075
105	6-phosphofructokinase, muscle type	PFKM	3.5021
106	Delta(14)-sterol reductase	TM7SF2	3.4938
107	Pre-mRNA-processing-splicing factor 8	PRPF8	3.4906
108	AP2-associated protein kinase 1	AAK1	3.4699
109	Surfeit locus protein 4	SURF4	3.453
110	Fatty aldehyde dehydrogenase	ALDH3A2	3.4464
111	Inositol-tetrakisphosphate 1-kinase	ITPK1	3.4435
112	N-acetylserotonin O-methyltransferase-like protein	ASMTL	3.4415
113	60S ribosomal protein L35a	RPL35A	3.4314
114	SPRY domain-containing protein 7	SPRYD7	3.4299
115	Serine/threonine-protein phosphatase 2A 56 kDa regulatory subunit delta isoform	PPP2R5D	3.423
116	60S ribosomal protein L8	RPL8	3.4216
117	ATP-binding cassette sub-family F member 2	ABCF2	3.4198
118	Selenocysteine-specific elongation factor	EEFSEC	3.4077
119	Myosin-14	MYH14	3.3967
120	Protein NipSnap homolog 1	NIPSNAP1	3.3914
121	60S ribosomal protein L24	RPL24	3.3829
122	ATP synthase subunit b, mitochondrial	ATP5F1	3.3809
123	UDP-glucose 6-dehydrogenase	UGDH	3.3751
124	DnaJ homolog subfamily C member 11	DNAJC11	3.3727
125	Cysteine dioxygenase type 1	CDO1	3.3683
126	Heat shock protein 75 kDa, mitochondrial	TRAP1	3.3625
127	Trifunctional enzyme subunit beta, mitochondrial;3-ketoacyl-CoA thiolase	HADHB	3.3616
128	V-type proton ATPase subunit B, brain isoform	ATP6V1B2	3.3592
129	Protein NipSnap homolog 2	GBAS	3.3543
130	ATPase family AAA domain-containing protein 3A;ATPase family AAA domain-containing protein 3B	ATAD3A;ATAD3B	3.3492
131	Transmembrane protein 43	TMEM43	3.3474
132	Translin-associated protein X	TSNAX	3.3342
133	Sideroflexin-4	SFXN4	3.3222
134	40S ribosomal protein S7	RPS7	3.311
135	6-phosphogluconolactonase	PGLS	3.3087
136	Clathrin heavy chain 1	CLTC	3.3033
137	NF-kappa-B-repressing factor	NKRF	3.3022
138	60S ribosomal protein L10;60S ribosomal protein L10-like	RPL10;RPL10L	3.2885
139	ADP-dependent glucokinase	ADPGK	3.2865

140	V-type proton ATPase catalytic subunit A	ATP6V1A	3.2815
141	Nucleoside diphosphate-linked moiety X motif 19, mitochondrial	NUDT19	3.28
142	Peroxisomal acyl-coenzyme A oxidase 1	ACOX1	3.2736
143	Pyruvate dehydrogenase E1 component subunit beta, mitochondrial	PDHB	3.2732
144	Phospholipase A-2-activating protein	PLAA	3.2711
145	Copine-3	CPNE3	3.2681
146	Serine/threonine-protein phosphatase 2A catalytic subunit alpha isoform;Serine/threonine-protein phosphatase 2A catalytic subunit beta isoform;Serine/threonine-protein phosphatase	PPP2CA;PPP2CB	3.2663
147	Ras-related protein Rab-8A	RAB8A	3.2638
148	Heterogeneous nuclear ribonucleoprotein U	HNRNPU	3.2621
149	Transmembrane protein 126A	TMEM126A	3.2569
150	Beta-adrenergic receptor kinase 1	ADRBK1	3.2536
151	60S ribosomal protein L10a	RPL10A	3.2527
152	2-oxoglutarate dehydrogenase, mitochondrial	OGDH	3.2522
153	Acyl-coenzyme A thioesterase 1;Acyl-coenzyme A thioesterase 2, mitochondrial	ACOT1;ACOT2	3.2431
154	MOSC domain-containing protein 1, mitochondrial	36951	3.2386
155	T-complex protein 1 subunit eta	CCT7	3.2384
156	ER membrane protein complex subunit 1	EMC1	3.2313
157	U5 small nuclear ribonucleoprotein 40 kDa protein	SNRNP40;DKFZp434D199	3.2294
158	Ribosomal protein 63, mitochondrial	MRP63	3.2288
159	Proteasome subunit alpha type-2	PSMA2	3.214
160	39S ribosomal protein L38, mitochondrial	MRPL38	3.2137
161	Nicalin	NCLN	3.2043
162	Copine-1	CPNE1	3.203
163	Isocitrate dehydrogenase [NADP];Isocitrate dehydrogenase [NADP], mitochondrial	IDH2	3.2006
164	CAD protein;Glutamine-dependent carbamoyl-phosphate synthase;Aspartate carbamoyltransferase;Dihydroorotase	CAD	3.1974
165	Lipopolysaccharide-responsive and beige-like anchor protein	LRBA	3.19
166	Caseinolytic peptidase B protein homolog	CLPB	3.1779
167	Very long-chain specific acyl-CoA dehydrogenase, mitochondrial	ACADVL	3.1758
168	Heterogeneous nuclear ribonucleoprotein R	HNRNPR	3.1634
169	C-1-tetrahydrofolate synthase, cytoplasmic;Methylenetetrahydrofolate dehydrogenase;Methenyltetrahydrofolate cyclohydrolase;Formyltetrahydrofolate synthetase;C-1-tetrahydrofolate synthase, cytoplasmic, N-terminally processed	MTHFD1	3.1597
170	Transmembrane protein 33	TMEM33	3.1543
171	WD40 repeat-containing protein SMU1	SMU1	3.1523
172	RNA-binding protein Raly	RALY	3.1433
173	Proliferation-associated protein 2G4	PA2G4	3.1405
174	Phosphate carrier protein, mitochondrial	SLC25A3	3.1393
175	Growth factor receptor-bound protein 10	GRB10	3.1391
176	Destrin	DSTN	3.1385
177	Cleavage and polyadenylation specificity factor subunit 1	CPSF1	3.1373
178	28S ribosomal protein S28, mitochondrial	MRPS28	3.1337
179	Ras-related protein Rab-13	RAB13	3.1302
180	Voltage-dependent anion-selective channel protein 2	VDAC2	3.1262
181	Bifunctional purine biosynthesis protein PURH;Phosphoribosylaminoimidazolecarboxamide formyltransferase;IMP cyclohydrolase	ATIC	3.1194

182	Cytoplasmic aconitate hydratase	ACO1;IRP1	3.1192
183	RNA polymerase-associated protein CTR9 homolog	CTR9	3.1184
184	Methionine--tRNA ligase, cytoplasmic	MARS	3.1183
185	DNA mismatch repair protein Mlh1	MLH1	3.1107
186	Putative phospholipase B-like 2;Putative phospholipase B-like 2 32 kDa form;Putative phospholipase B-like 2 45 kDa form	PLBD2	3.1057
187	Mitochondrial 2-oxoglutarate/malate carrier protein	SLC25A11	3.1049
188	NADPH--cytochrome P450 reductase	POR	3.0977
189	Heterogeneous nuclear ribonucleoprotein A0	HNRNPA0	3.0976
190	Tryptophan--tRNA ligase, cytoplasmic;T1-TrpRS;T2-TrpRS	WARS	3.0964
191	Signal transducer and activator of transcription 1-alpha/beta	STAT1	3.0958
192	E3 SUMO-protein ligase RanBP2;Putative peptidyl-prolyl cis-trans isomerase	RANBP2	3.091
193	Glutamine--tRNA ligase	QARS	3.0906
194	Phosphatidylinositol transfer protein alpha isoform	PITPNA	3.0877
195	Mitochondrial import inner membrane translocase subunit Tim23;Putative mitochondrial import inner membrane translocase subunit Tim23B	TIMM23;TIMM23B	3.075
196	60S ribosomal protein L36	RPL36	3.0734
197	ADP/ATP translocase 2;ADP/ATP translocase 2, N-terminally processed	SLC25A5	3.0689
198	Cytochrome b-c1 complex subunit 1, mitochondrial	UQCRC1	3.0672
199	40S ribosomal protein S4, X isoform	RPS4X	3.0661
200	5-3 exoribonuclease 2	XRN2	3.0628
201	Core histone macro-H2A.1;Histone H2A	H2AFY	3.0615
202	Purine nucleoside phosphorylase	PNP	3.059
203	Biliverdin reductase A	BLVRA	3.0586
204	Heterogeneous nuclear ribonucleoprotein L	HNRNPL	3.0539
205	X-ray repair cross-complementing protein 5	XRCC5	3.0538
206	60S ribosomal protein L5	RPL5	3.0527
207	Glutathione S-transferase kappa 1	GSTK1	3.0498
208	Ubiquitin-like modifier-activating enzyme 1	UBA1	3.0411
209	26S proteasome non-ATPase regulatory subunit 10	PSMD10	3.0399
210	Lysosomal alpha-glucosidase;76 kDa lysosomal alpha-glucosidase;70 kDa lysosomal alpha-glucosidase	GAA	3.0394
211	Moesin	MSN	3.0389
212	Fatty acid synthase;[Acyl-carrier-protein] S-acetyltransferase;[Acyl-carrier-protein] S-malonyltransferase;3-oxoacyl-[acyl-carrier-protein] synthase;3-oxoacyl-[acyl-carrier-protein] reductase;3-hydroxyacyl-[acyl-carrier-protein] dehydratase;Enoyl-[acyl-carrier-protein] reductase;Oleoyl-[acyl-carrier-protein] hydrolase	FASN	3.0364
213	39S ribosomal protein L19, mitochondrial	MRPL19	3.0353
214	Elongation factor 1-gamma	EEF1G	3.0243
215	Pro-cathepsin H;Cathepsin H mini chain;Cathepsin H;Cathepsin H heavy chain;Cathepsin H light chain	CTSH	3.0235
216	Cold shock domain-containing protein E1	CSDE1	3.0217
217	Phosphatidylglycerophosphatase and protein-tyrosine phosphatase 1	PTPMT1	3.0195
218	Armadillo repeat protein deleted in velo-cardio-facial syndrome	ARVCF	3.0176
219	Trifunctional enzyme subunit alpha, mitochondrial;Long-chain enoyl-CoA hydratase;Long chain 3-hydroxyacyl-CoA dehydrogenase	HADHA	3.0157
220	26S proteasome non-ATPase regulatory subunit 14	PSMD14	3.0147
221	Striatin-3	STRN3	3.0115

222	Beta-hexosaminidase;Beta-hexosaminidase subunit alpha	HEXA	3.009
223	Actin-like protein 6A	ACTL6A	3.0043
224	Regulator of chromosome condensation	RCC1	3.0034
