# Supplementary material for: The long noncoding RNA HORAS5 mediates castration‐resistant prostate cancer survival by activating the androgen receptor transcriptional program
Source: Mol Oncol. 2019 Mar 5;13(5):1121–36. doi: 10.1002/1878-0261.12471 (PMC6487714; doi:10.1002/1878-0261.12471)
Supplement: Supplementary file 16 — Table S4. List of proteins that are down‐regulated (FC≤3) upon HORAS5 knockdown in LNCaP cells. [file MOL2-13-1121-s016.pdf]

Supplemental Table 4: List of proteins that are down-regulated (FC<=3) upon HORAS5 knockdown in LNCaP cells

Rank#	Protein names	Gene names	Fold Change siHORAS5/siNC (normalized)
1	Core-binding factor subunit beta	CBFB	0.033616
2	Vesicle-associated membrane protein 1	VAMP1	0.1152
3	DnaJ homolog subfamily C member 9	DNAJC9	0.12095
4	PCNA-associated factor	KIAA0101	0.18324
5	High mobility group protein B2	HMGB2	0.19629
6	Protein CASC4	CASC4	0.20482
7	Short coiled-coil protein	SCOC	0.21613
8	Frataxin, mitochondrial;Frataxin intermediate form;Frataxin(56-210);Frataxin(78-210);Frataxin mature form	FXN	0.23828
9	Reticulocalbin-1	RCN1	0.2383
10	Uncharacterized protein C1orf122	C1orf122	0.24014
11	Tumor protein D54	TPD52L2	0.2617
12	U6 snRNA-associated Sm-like protein LSm3	LSM3	0.26658
13	Translationally-controlled tumor protein	TPT1	0.2675
14	Stathmin	STMN1	0.27899
15	Periphilin-1	PPHLN1	0.28428
16	Putative monooxygenase p33MONOX	KIAA1191	0.28739
17	Lysine-specific demethylase 5B	JARID1B;KDM5B	0.29453
18	Wiskott-Aldrich syndrome protein family member 2	WASF2	0.30279
19	Lamina-associated polypeptide 2, isoforms beta/gamma;Thymopoietin;Thymopentin	TMPO	0.30282
20	NFATC2-interacting protein	NFATC2IP	0.30292
21	Alpha-taxilin	TXLNA	0.30377
22	Eukaryotic translation initiation factor 4H	EIF4H	0.30475
23	Specifically androgen-regulated gene protein	SARG	0.30749
24	RNA polymerase-associated protein LEO1	LEO1	0.31107
25	Heme-binding protein 2	HEBP2	0.31374
26	Leucine-rich repeat flightless-interacting protein 1	LRRFIP1	0.31449
27	Chromatin assembly factor 1 subunit A	CHAF1A	0.31898
28	Translocon-associated protein subunit alpha	SSR1	0.3191
29	Transcription cofactor vestigial-like protein 4	VGLL4	0.32203
30	Ubiquilin-4	UBQLN4	0.32364
31	Alpha-2-macroglobulin receptor-associated protein	LRPAP1	0.3255
32	TATA element modulatory factor	TMF1	0.32578
33	E3 ubiquitin-protein ligase RNF181	RNF181	0.32802
34	Cyclin-dependent kinase inhibitor 1	CDKN1A	0.32994
