# Supplementary material for: The long noncoding RNA HORAS5 mediates castration‐resistant prostate cancer survival by activating the androgen receptor transcriptional program
Source: Mol Oncol. 2019 Mar 5;13(5):1121–36. doi: 10.1002/1878-0261.12471 (PMC6487714; doi:10.1002/1878-0261.12471)
Supplement: Supplementary file 17 — Table S5. Top biological concepts significantly associated with genes downregulated upon HORAS5 knockdown. [file MOL2-13-1121-s017.pdf]

Supplemental Table 5: Top biological concepts significantly associated with genes downregulated upon HORAS5 knockdown.

Biological Function	P value	Odds Ratio
RNA polymerase II-transcription factor activity	0.001	15.1
Unfolded protein binding	0.002	12.5
Protein folding	0.005	9.4
Cell proliferation	0.008	8
