# Supplementary material for: The long noncoding RNA HORAS5 mediates castration‐resistant prostate cancer survival by activating the androgen receptor transcriptional program
Source: Mol Oncol. 2019 Mar 5;13(5):1121–36. doi: 10.1002/1878-0261.12471 (PMC6487714; doi:10.1002/1878-0261.12471)
Supplement: Supplementary file 18 — Table S6. List of pre‐designed Taqman probes used for qPCR analyses. [file MOL2-13-1121-s018.pdf]

Supplemental Table 6: List of pre-designed Taqman probes used for qPCR analyses

(ThermoFisher, Cat#s for all 4331182)

**Gene ID**	**Assay ID**
GAPDH	Hs02786624_g1
HPRT1	Hs02800695_m1
5s rRNA	Hs03682751_gH
HORAS5 (Long Variant)	Hs00863167_g1
HORAS5 (Short Variant)	Hs00862162_g1
PCAT4	Hs00328566_m1
snoRNA55	Hs03298696_s1
KIAA0101	Hs00207134_m1
STMN1	Hs01027515_gH
KDM5B	Hs00981910_m1

Custom Taqman probes (Cat# 4441114)

RP11-945A11.1
AF131217.1
AP001604.3
